# On coefficients of Poincaré series and single-valued periods of modular forms

**DOI:** 10.1007/s40687-020-00232-5

**Published:** 2020-11-05

**Authors:** Tiago J. Fonseca

**Affiliations:** Mathematical Institute, University of Oxford, Andrew Wiles Building, Radcliffe Observatory Quarter, Woodstock Road, Oxford, OX2 6GG UK

**Keywords:** Poincaré series, Fourier coefficients, Single-valued periods, Weakly holomorphic modular forms, Modular motives, 11F30, 11F67, 32G20, 14J15

## Abstract

We prove that the field generated by the Fourier coefficients of weakly holomorphic Poincaré series of a given level $\varGamma _0(N)$ and integral weight $k\ge 2$ coincides with the field generated by the single-valued periods of a certain motive attached to $\varGamma _0(N)$. This clarifies the arithmetic nature of such Fourier coefficients and generalises previous formulas of Brown and Acres–Broadhurst giving explicit series expansions for the single-valued periods of some modular forms. Our proof is based on Bringmann–Ono’s construction of harmonic lifts of Poincaré series.

## Introduction

### Fourier coefficients of Poincaré series

Let $N\ge 1$ be an integer and consider Hecke’s congruence subgroup

$$\begin{aligned} \varGamma _0(N) =\left\{ \left( \begin{array}{cc} a &{} b \\ c &{} d \end{array}\right) \in {\mathrm {SL}}_2({\mathbb {Z}}) \text { ; } c \equiv 0 \mod N \right\} \le {\mathrm {SL}}_2({\mathbb {Z}})\text {.} \end{aligned}$$

For every integer *m* and every even integer $k> 2$, we define a *Poincaré series* (cf. [12, 28])

1 $$\begin{aligned} P_{m,k,N}(\tau ) = \sum _{\gamma \, \in \, \varGamma _\infty \backslash \varGamma _0(N)}\frac{e^{2\pi i m \gamma \cdot \tau }}{j(\gamma ,\tau )^k}\text {, }\qquad \tau \in {\mathbb {H}}\text {,} \end{aligned}$$

where ${\mathbb {H}}$ denotes the Poincaré half-plane with its usual $\mathrm {SL}_2({\mathbb {R}})$-action $\gamma \cdot \tau = (a\tau +b)(c\tau +d)^{-1}$, $j(\gamma ,\tau ) = c\tau +d$ is the factor of automorphy, and $\varGamma _{\infty }\le \varGamma _0(N)$ is the stabiliser of the cusp at infinity, given by the condition $c=0$.

The series (1) converges absolutely and uniformly on compact subsets of ${\mathbb {H}}$ and defines a weakly holomorphic modular form of weight *k* and level $\varGamma _0(N)$, meaning that

$$\begin{aligned} P_{m,k,N}(\gamma \cdot \tau ) = j(\gamma ,\tau )^kP_{m,k,N}(\tau ) \end{aligned}$$

for every $\gamma \in \varGamma _0(N)$, and that $P_{m,k,N}$ is holomorphic on ${\mathbb {H}}$, and meromorphic at the cusps. When $m=0$, we obtain an Eisenstein series; this case will often be excluded from our discussion. Following Petersson [27], we shall also consider Poincaré series in weight $k=2$, which are defined via a variation of Hecke’s trick.

To a certain extent, the theory of cusp forms reduces to the study of Poincaré series of positive index *m*, as it is well known that $P_{m,k,N}$, for $m>0$, generate the finite-dimensional ${\mathbb {C}}$-vector space of cusp forms $S_{k}(\varGamma _0(N))$. A natural question is then to understand the Fourier coefficients $a_n(P_{m,k,N})$, defined by

$$\begin{aligned} P_{m,k,N} = \sum _{n\ge 1} a_n(P_{m,k,N}) q^n\text {, } \qquad q=e^{2\pi i \tau }\text {.} \end{aligned}$$

When the index *m* is positive, a classical computation yields the formulas

2 $$\begin{aligned} a_n(P_{m,k,N}) = \delta _{m,n}+ 2\pi (-1)^{\frac{k}{2}}\left( \frac{n}{m} \right) ^{\frac{k-1}{2}}\sum _{c\ge 1\text {, } N \mid c}\frac{K(m,n;c)}{c}J_{k-1}\left( \frac{4\pi \sqrt{mn}}{c} \right) \end{aligned}$$

for every $n\ge 1$, where $K(a,b;c) = \sum _{x \in ({\mathbb {Z}}/c{\mathbb {Z}})^{\times }}e^{\frac{2\pi i}{c}(ax + bx^{-1})}$ is a Kloosterman sum and $J_{k-1}$ is the *J*-Bessel function of order $k-1$; when the index is negative, we have

$$\begin{aligned} P_{-m,k,N} = q^{-m} + \sum _{n\ge 1} a_{n}(P_{-m,k,N})q^n\text {,}\qquad q=e^{2\pi i \tau }\text {,} \end{aligned}$$

with

3 $$\begin{aligned} a_n(P_{-m,k,N}) = 2\pi (-1)^{\frac{k}{2}}\left( \frac{n}{m} \right) ^{\frac{k-1}{2}}\sum _{c\ge 1\text {, } N \mid c}\frac{K(-m,n;c)}{c}I_{k-1}\left( \frac{4\pi \sqrt{mn}}{c} \right) \end{aligned}$$

for every $n\ge 1$, where $I_{k-1}$ is an *I*-Bessel function of order $k-1$.

The expressions (2) and (3) play a prominent role in questions involving growth estimates of Fourier coefficients of modular forms (see [32]), but are unsatisfying from an algebraic point of view. Although the real numbers $a_n(P_{m,k,N})$, for positive *m*, are expected to be transcendental whenever they are not zero^1^, the Fourier coefficients of Poincaré series of *negative* index, $a_n(P_{-m,k,N})$, can satisfy remarkable rationality properties.

For instance, a classical result of Petersson [27] and Rademacher [29] yields

$$\begin{aligned} P_{-1,2,1} = -Dj = q^{-1} - 196884 q - 42987520 q^2 - 2592899910 q^3 - \cdots \text {,} \end{aligned}$$

where *j* denotes Klein’s modular invariant and *D* is the derivation $\frac{1}{2\pi i}\frac{d}{d\tau } = q\frac{d}{dq}$. Numerical experimentation provides other examples of Poincaré series with integral Fourier coefficients, such as

$$\begin{aligned} P_{-2,6,4} = q^{-2} -35q^2 + 4096q^4 - 97686q^6 + \cdots \end{aligned}$$

and

$$\begin{aligned} P_{-1,4,9} = q^{-1} + 2q^2 -49q^5 + 48q^8 + 771q^{11} - \cdots \text {,} \end{aligned}$$

but most Poincaré series with negative index are also expected to have transcendental Fourier coefficients.

The present work is based on the observation that all of the above rationality phenomena can be ‘explained’ by cohomology. The case of $P_{-1,4,9}$ was proved by Bruinier et al. [10] using the theory of harmonic Maass forms, the key ingredient being the fact that the unique normalised newform in $S_{4}(\varGamma _0(9))$ has CM. In the end of [11], Candelori briefly discusses how to reformulate this result in terms of a certain cohomology group with local coefficients over the modular curve $X_1(9)$.

This hints to the fact that Fourier coefficients of Poincaré series, in general, are cohomological invariants. In this article, we show that $a_n(P_{m,k,N})$, $m\in {\mathbb {Z}}\setminus \{0\}$, are all rational expressions in *periods* of the modular motive $H^1_{\mathrm {cusp}}({\mathcal {Y}}_0(N), V_k)$ (see Sect. 0.2 below). These periods, in turn, are related to the classical periods $\omega ^+$ and $\omega ^-$ of cusp forms, and also to their ‘quasi-periods’ $\eta ^+$ and $\eta ^-$, which have recently been introduced by Brown and Hain [8]. As we shall explain below, our result is actually more precise: it identifies the ${\mathbb {Q}}$-extension generated by all the Fourier coefficients $a_n(P_{m,k,N})$, for $m \in {\mathbb {Z}}\setminus \{0\}$, with the ${\mathbb {Q}}$-extension generated by a certain subclass of periods of $H^1_{\mathrm {cusp}}({\mathcal {Y}}_0(N), {\mathcal {V}}_k)$, the *single-valued periods*, in the terminology of Brown [5, 7]. A first manifestation of identities relating Fourier coefficients of Poincaré series and single-valued periods appears already in the work of Brown [6]. Subsequent work of Broadhurst and Acres and Broadhurst [1] provided many other explicit examples of such identities, under the guise of ‘Rademacher sums’ (see below). Our work can also be regarded as a generalisation of Brown’s and Acres–Broadhurst’s formulas.

### Single-valued periods of modular motives

Given a smooth algebraic variety *X* defined over a number field $K\subset {\mathbb {C}}$ and an integer $i\ge 0$, there is a canonical comparison isomorphism [19]

$$\begin{aligned} \mathrm {comp}: H_{\mathrm {dR}}^i(X)\otimes _K {\mathbb {C}} {\mathop {\rightarrow }\limits ^{\sim }} H_{\mathrm {B}}^i(X)\otimes _{{\mathbb {Q}}}{\mathbb {C}} \end{aligned}$$

given by ‘integration of differential forms’, where $H_{\mathrm {dR}}^i(X)$ denotes the *i*th algebraic de Rham cohomology with coefficients in *K* and $H^i_{\mathrm {B}}(X)$ denotes the *i*th Betti cohomology of *X*, defined as the dual ${\mathbb {Q}}$-vector space of the *i*th singular homology $H_i(X({\mathbb {C}});{\mathbb {Q}}) = H_i(X({\mathbb {C}});{\mathbb {Z}})\otimes _{{\mathbb {Z}}}{\mathbb {Q}}$ of the complex manifold $X({\mathbb {C}})$. A *period* [23] of $H^i(X)$ is a complex number of the form

$$\begin{aligned} \langle \sigma ,\mathrm {comp}(\alpha ) \rangle = \int _{\sigma }\alpha \in {\mathbb {C}} \end{aligned}$$

for some $\alpha \in H^i_{\mathrm {dR}}(X)$ and $\sigma \in H_i(X({\mathbb {C}});{\mathbb {Q}}) = H^i_{\mathrm {B}}(X)^{\vee }$.

When $K\subset {\mathbb {R}}$, the continuous involution $X({\mathbb {C}}) \rightarrow X({\mathbb {C}})$ given by complex conjugation induces a ${\mathbb {Q}}$-linear involution

$$\begin{aligned} F_{\infty }: H^i_{\mathrm {B}}(X) \rightarrow H^i_{\mathrm {B}}(X)\text {,} \end{aligned}$$

sometimes called the ‘real Frobenius’. By transport of structures, we obtain an involution $\mathrm {sv}:=\mathrm {comp}^{-1} \circ (F_{\infty }\otimes \mathrm {id}) \circ \mathrm {comp}$ on $H^i_{\mathrm {dR}}(X)\otimes _K{\mathbb {C}}$, which can actually be shown to be defined over ${\mathbb {R}}$; this is the *single-valued involution*:

$$\begin{aligned} \mathrm {sv}: H^i_{\mathrm {dR}}(X)\otimes _K {\mathbb {R}} \rightarrow H^i_{\mathrm {dR}}(X)\otimes _K {\mathbb {R}}\text {.} \end{aligned}$$

A *single-valued period* [7] of $H^i(X)$ is a real number of the form

$$\begin{aligned} \langle \varphi , \mathrm {sv}(\alpha )\rangle \in {\mathbb {R}} \end{aligned}$$

for some $\alpha \in H^i_{\mathrm {dR}}(X)$ and $\varphi \in H^i_{\mathrm {dR}}(X)^{\vee }$.

Single-valued periods can be understood in terms of periods as follows. Given a period matrix *P* of $H^i(X)$, that is, a matrix of $\mathrm {comp}$ in a *K*-basis of $H_{\mathrm {dR}}^i(X)$ and a ${\mathbb {Q}}$-basis of $H_{\mathrm {B}}^i(X)$, a single-valued period matrix is given by

4 $$\begin{aligned} S = P^{-1}{\overline{P}} = P^{-1}F_{\infty }P\text {.} \end{aligned}$$

The terminology ‘single-valued period’ has its origin in the theory of period functions (i.e. periods of families of algebraic varieties); we refer to [7] for further details and motivation.

#### Example 1

Consider the elliptic curve *E* over ${\mathbb {Q}}$ given by the equation

$$\begin{aligned} y^2 + y = x^3 - x^2 -10x -20\text {.} \end{aligned}$$

A basis for its first de Rham cohomology $H^1_{\mathrm {dR}}(E)$ is represented by $(\omega ,\eta ) = (\frac{dx}{2y +1}, x\frac{dx}{2y+1})$. Given a oriented basis $(\gamma _1,\gamma _2)$ of $H_1(E({\mathbb {C}});{\mathbb {Z}})$, we obtain a period matrix

$$\begin{aligned} P = \left( \begin{array}{cc} \omega _1 &{} \eta _1 \\ \omega _2 &{} \eta _2 \end{array}\right) = \left( \begin{array}{cc} \int _{\gamma _1}\omega &{} \int _{\gamma _1}\eta \\ \int _{\gamma _2}\omega &{} \int _{\gamma _2}\eta \end{array}\right) \in {\mathrm {GL}}_2({\mathbb {C}}) \end{aligned}$$

satisfying $\det P = 2\pi i$ (Legendre’s period relation). The single-valued period matrix with respect to the basis $([\omega ],[\eta ])$ is then given by

$$\begin{aligned} S = \frac{1}{2\pi i}\left( \begin{array}{cc} {\overline{\omega }}_1\eta _2-{\overline{\omega }}_2\eta _1 &{} {\overline{\eta }}_1\eta _2 - \eta _1{\overline{\eta }}_2 \\ \omega _1{\overline{\omega }}_2 - {\overline{\omega }}_1\omega _2 &{} \omega _1{\overline{\eta }}_2-\omega _2{\overline{\eta }}_1 \end{array} \right) \in {\mathrm {GL}}_2({\mathbb {R}})\text {.} \end{aligned}$$

It does not depend on the choice of $(\gamma _1,\gamma _2)$, and necessarily satisfies $S^2=\mathrm {id}$ and $\text {Tr }S = 0$. Numerically, we can compute a period lattice basis $\omega _1,\omega _2$ in Sage [31] and obtain the quasi-periods $\eta _1,\eta _2$ by integrating the Weierstrass $\wp$ function (here, $(\omega ,\eta ) = (dz, \wp (z)dz + \frac{1}{3}dz)$). We get

$$\begin{aligned} P = \left( \begin{array}{cc} 1.269209... &{} -2.214333... \\ 0.634604... + i 1.458816... &{} -1.107166... + i 2.405338... \end{array}\right) \end{aligned}$$

and

$$\begin{aligned} S = \left( \begin{array}{cc} -0.028238... &{} -1.695389... \\ -0.589364... &{} 0.028238... \end{array}\right) \text {.} \end{aligned}$$

More generally, we can talk about periods and single-valued periods of motives, which can be thought as compatible systems of realisations (de Rham, Betti, étale, etc.) of ‘geometric origin’. For instance, the classical periods $\omega ^+$, $\omega ^-$ of a normalised Hecke cusp form $f \in S_{k+2}(\varGamma _0(1))$ ([12, 25] Chapter 11) are defined as real numbers for which there is a decomposition

$$\begin{aligned} \int _{0}^{i\infty }f(\tau )(X-\tau Y)^kd\tau = \omega ^+ P^+ + i \omega ^-P^-\text {, }\qquad P^{\pm } \in K_f[X,Y]\text {,} \end{aligned}$$

with $P^+$ (resp. $P^-$) an even (resp. odd) homogeneous polynomial in *X*, *Y*. Here, $K_f={\mathbb {Q}}(a_n(f)\text { ; } n \ge 1)$ is the totally real number field generated by the Fourier coefficients of *f*. Up to a power of $2\pi i$, the numbers $\omega ^+$ and $\omega ^-$ are periods of a pure motive $H_f$, occurring in the cohomology of a Kuga–Sato variety [35].

In practice, instead of going to the Kuga–Sato variety, it is convenient to stay at the modular curve and to work with cohomology with local coefficients. For every integer $N\ge 1$ we denote by ${\mathcal {Y}}_0(N)$ the moduli stack over ${\mathbb {Q}}$ classifying elliptic curves endowed with a cyclic subgroup of order *N*. Its analytification is isomorphic to the orbifold quotient $\varGamma _0(N)\backslash \! \backslash {\mathbb {H}}$, and its corresponding coarse moduli space is isomorphic to the usual open modular curve $Y_0(N)$ over ${\mathbb {Q}}$. Let ${\mathcal {E}}$ be the universal elliptic curve over ${\mathcal {Y}}_0(N)$.

Explicitly, there is a (mixed) motive $H^1({\mathcal {Y}}_0(N),V_k)$ over ${\mathbb {Q}}$ whose de Rham realisation is given by

$$\begin{aligned} H^1({\mathcal {Y}}_0(N),V_k)_{\mathrm {dR}} = H^1_{\mathrm {dR}}({\mathcal {Y}}_0(N), {\mathcal {V}}_k)\text {,} \end{aligned}$$

where ${\mathcal {V}}_k$ is the vector bundle $\mathrm {Sym}^k H_{\mathrm {dR}}^1({\mathcal {E}}/{\mathcal {Y}}_0(N))$ endowed with the Gauss–Manin connection. This cohomology group admits a description in terms of weakly holomorphic modular forms (cf. [8, 11, 13, 22, 34]): there is a canonical exact sequence of ${\mathbb {Q}}$-vector spaces

5 $$\begin{aligned} 0 \rightarrow M_{-k}^!(\varGamma _0(N); {\mathbb {Q}}) {\mathop {\rightarrow }\limits ^{D^{k+1}}} M_{k+2}^!(\varGamma _0(N);{\mathbb {Q}}) \rightarrow H^1_{\mathrm {dR}}({\mathcal {Y}}_0(N), {\mathcal {V}}_k) \rightarrow 0\text {,} \end{aligned}$$

where $M^!_{r}(\varGamma _0(N);{\mathbb {Q}})$ denotes the space of weakly holomorphic modular forms of weight *r* and level $\varGamma _0(N)$ with rational Fourier coefficients at infinity, and $D^{k+1}$ is the ‘Bol operator’. The Betti realisation of $H^1({\mathcal {Y}}_0(N),V_k)$ is isomorphic to the classical group cohomology of $\varGamma _0(N)$ considered by Eichler and Shimura (see Sect. 6 below).

Motives of cusp forms are found in the weight $k+1$ part of $H^1({\mathcal {Y}}_0(N),V_k)$, which is a polarisable pure motive $H_{\mathrm {cusp}}^1({\mathcal {Y}}_0(N),V_k)$ of Hodge type $\{(k+1,0),(0,k+1)\}$ whose realisations are given by cuspidal (or parabolic) cohomology. The single-valued periods of $H_{\mathrm {cusp}}^1({\mathcal {Y}}_0(N),V_k)$ are the main object of study of this paper. These include, up to a power of $2\pi i$, the Petersson norms of modular forms of weight $k+2$ and level $\varGamma _0(N)$, but also some non-standard quantities, as we shall see below.

### Single-valued periods and coefficients of Poincaré series

Our main result is the following.

#### Theorem 1

(cf. Theorem 8 below) Let $k\ge 0$ and $N\ge 1$ be integers, with *k* even, and let $S = (s_{ij})_{1\le i,j\le r}$ be a single-valued period matrix with respect to a ${\mathbb {Q}}$-basis of $H_{\mathrm {cusp}}^1({\mathcal {Y}}_0(N),V_k)_{\mathrm {dR}}$. Then,

$$\begin{aligned} {\mathbb {Q}}(s_{ij} \text { ; } 1\le i,j\le r) = {\mathbb {Q}}(a_n(P_{m,k+2,N}) \text { ; for all } n\ge 1\text { and } m\ne 0)\text {.} \end{aligned}$$

As an immediate corollary of the above theorem, we deduce that the field generated by Fourier coefficients of Poincaré series of positive and negative index ${\mathbb {Q}}(a_n(P_{m,k+2,N}) \text { ; } n\ge 1\text {, } m\ne 0)$ is finitely generated over ${\mathbb {Q}}$. In the degenerate case where $S_{k+2}(\varGamma _0(N))=0$ (or, equivalently, $H^1_{\mathrm {cusp}}({\mathcal {Y}}_0(N),V_k) = 0$), there are no single-valued periods; in other words, *S* should be taken as the empty matrix, and the statement amounts to asserting that the Fourier coefficients of the corresponding Poincaré series are all rational. This is the case of $P_{-1,2,1}$ considered above.

Apart from terminology, that Fourier coefficients of Poincaré series of *positive* index *m* can be written as rational expressions in single-valued periods of $H_{\mathrm {cusp}}^1({\mathcal {Y}}_0(N),V_k)$ is in fact a classical statement following from Petersson’s formula: for every $f \in S_{k+2}(\varGamma _0(N))$,

6 $$\begin{aligned} \begin{aligned} (f,P_{m,k+2,N})_{\mathrm {Pet}}&:=\int _{\varGamma _0(N)\backslash {\mathbb {H}}}f(x+iy)\overline{P_{m,k+2,N}(x+iy)}y^{k+2}\frac{dx\wedge dy}{y^2}\\&= \frac{k!}{(4\pi m)^{k+1}}a_m(f)\text {.} \end{aligned} \end{aligned}$$

The main novelty in Theorem 1 is the case of negative index.

#### Example 2

The elliptic curve *E* of Example 1 is isomorphic to the modular curve $X_0(11)$. In particular, since $H^1(X_0(11)) = H^1_{\mathrm {cusp}}({\mathcal {Y}}_0(11),V_0)$, Theorem 1 implies that the Fourier coefficients of $P_{m,2,11}$ are rational expressions in the single-valued periods of $H^1(E)$ for every $m \in {\mathbb {Z}}\setminus \{0\}$. For instance, we have

$$\begin{aligned} a_1(P_{1,2,11}) =-\frac{2\pi i}{\omega _1{\overline{\omega }}_2 - {\overline{\omega }}_1\omega _2} = 1.696742... \end{aligned}$$

and

$$\begin{aligned} a_{1}(P_{-1,2,11}) = \frac{{\overline{\omega }}_1\eta _2-{\overline{\omega }}_2\eta _1}{\omega _1{\overline{\omega }}_2 - {\overline{\omega }}_1\omega _2} - 1 = -0.952086... \end{aligned}$$

This can be checked numerically using formulas (2) and (3). The expression for $a_1(P_{1,2,11})$ is classical: it follows from (6), which shows that $a_1(P_{1,2,11})^{-1} = 4\pi (f,f)_{\mathrm {Pet}}$, with *f* the unique normalised cusp form in $S_2(\varGamma _0(11))$.

In general, when $\dim S_{k+2}(\varGamma _0(N))=1$ (see Table 1 in Sect. 8 below), that is, when the motive $H^1_{\mathrm {cusp}}({\mathcal {Y}}_0(N),V_k)$ is of rank 2, we prove a refined version of Theorem 1. In this case, let us denote by

$$\begin{aligned} \left( \begin{array}{cc} s_{11} &{} s_{12} \\ s_{21} &{} s_{22} \end{array}\right) \end{aligned}$$

the single-valued period matrix with respect to a symplectic basis ([*f*], [*g*]) of $H^1_{\mathrm {cusp}}({\mathcal {Y}}_0(N),V_k)_{\mathrm {dR}}$, induced by a cusp form *f* and a weakly holomorphic modular form *g* (see (5)).

#### Theorem 2

(cf. Proposition 8 below) With the above notation, for every integer $m\ge 1$, there exists $h_m \in M_{-k}^!(\varGamma _0(N);{\mathbb {Q}})$, depending on *f* and *g*, such that, for every $n\ge 1$,

$$\begin{aligned} a_n(P_{m,k+2,N}) = -\frac{k!}{m^{k+1}}a_m(f)a_n(f)\frac{1}{s_{21}} \end{aligned}$$

and

$$\begin{aligned} a_n(P_{-m,k+2,N}) = \frac{k!}{m^{k+1}}a_m(f)a_n(f)\frac{s_{11}}{s_{21}} + r_{m,n} \text {,} \end{aligned}$$

where

$$\begin{aligned} r_{m,n} = \frac{k!}{m^{k+1}}a_m(f)a_n(g) + n^{k+1}a_n(h_m) \in {\mathbb {Q}}\text {.} \end{aligned}$$

From the relations $S^2 = \mathrm {id}$ and $\text {Tr }S = 0$, we conclude that the entries $s_{12}$ and $s_{22}$ of *S* are determined by $s_{11}$ and $s_{21}$, so that the above result indeed refines Theorem 1 in the rank 2 case.

The above theorem also explains some rationality phenomena. For instance, when $a_m(f)=0$, then the above formulas imply that $P_{m,k+2,N}$ vanishes identically and that $a_n(P_{-m,k+2,N})$ is a rational number for all $n\ge 1$. When the unique normalised cusp form in $S_{k+2}(\varGamma _0(N))$ has CM, the motive $H^1_{\mathrm {cusp}}({\mathcal {Y}}_0(N),V_k)$ acquires extra endomorphisms after base change to a CM field ([33] Chapter V), and we can deduce from this that the quotient of single-valued periods $s_{11}/s_{21}$ is a rational number (cf. Example 4 below); thus, $P_{-m,k+2,N}$ has rational Fourier coefficients.

Theorem 2 generalises Brown’s Corollary 1.4 in [6], concerning level 1 and weight 12. It also provides a proof for the formulas in ‘genus 1’ of Acres and Broadhurst [1] found by numerical experimentation. To see this, one must remark that the ‘Rademacher sums’ of [1] are coefficients of Poincaré series after the action of the Fricke involution (cf. [24] Proposition 5.4). In general, Theorem 1 gives a conceptual explanation for the identities involving periods and quasi-periods of modular forms found by Broadhurst and Acres–Broadhurst (see also the formulas (39) below).

#### Remark 1

In higher rank, after base change to an appropriate number field *K*, we can split $H^1_{\mathrm {cusp}}({\mathcal {Y}}_0(N),V_k)$ by using Hecke operators; this leads to simple formulas (with coefficients in *K*) akin to the rank 2 case. This is worked out in Sect. 10 in the case where there are no old forms, such as in level 1.

Our proof method relies on the theory of harmonic Maass forms and in its relation with the single-valued involution as explained by Brown [6]. We summarise this relation in the following theorem, which reformulates and generalises Brown’s results (cf. Candelori [11]).

#### Theorem 3

(cf. Corollary 4 below) The following diagram of ${\mathbb {C}}$-vector spaces commutes: 
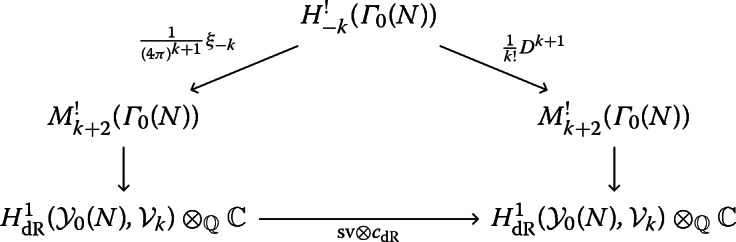
 where the vertical maps are induced by (5), and $c_{\mathrm {dR}}$ denotes complex conjugation on coefficients.

Here, $H_{-k}^!(\varGamma _0(N))$ denotes the space of harmonic Maass forms of manageable growth of weight $-k$ and level $\varGamma _0(N)$ (see Sect. 7 below), and $\xi _{-k}$ and $D^{k+1}$ are the differential operators considered in [10]. Let us remark that Theorem 3 clarifies a number of constructions in the theory of weakly holomorphic modular forms and harmonic Maass forms. For instance, using the above diagram one can show that the ‘regularised Petersson inner product’ of [3] is induced by the usual Hermitian pairing on $H^1_{\mathrm {dR}}({\mathcal {Y}}_0(N),{\mathcal {V}}_k)\otimes _{{\mathbb {Q}}} {\mathbb {C}}$ given by the polarisation of its Hodge structure (see Remark 11 below); this immediately implies, among other properties, the compatibility of the regularised inner product with Hecke operators.

By combining Theorem 3 with a theorem of Bringmann and Ono on the harmonic lifts of Poincaré series [2], we deduce the following result, which is the main ingredient in the proof of Theorems 1 and 2.

#### Proposition 1

(cf. Proposition 7 below) For every integer $m\ne 0$, the image of $P_{m,k+2,N}$ in $H^1_{\mathrm {cusp}}({\mathcal {Y}}_0(N),V_k)_{\mathrm {dR}}\otimes _{{\mathbb {Q}}} {\mathbb {R}}$ satisfies

$$\begin{aligned} \mathrm {sv}([P_{m,k+2,N}]) = - [P_{-m,k+2,N}]\text {.} \end{aligned}$$

### Organisation of the article

In Sects. 0 and 1 we outline the basic formalism concerning single-valued periods. As in [7], we avoid explicit mention of a category of motives by working in a suitable category of realisations ${\mathcal {H}}$, which is sufficient for our purposes. We then focus on the case of pure polarisable objects of ${\mathcal {H}}$ of Hodge type $\{(n,0),(0,n)\}$, as ‘motives of modular forms’ are of this type. Our main result here is Proposition 2, which explicitly describes the algebraic relations the single-valued period matrix of such an object must satisfy.

Sections 2 and 3 concern the theory of weakly holomorphic modular forms; their main purpose is to set up notation. We first introduce several spaces of weakly holomorphic modular forms for the group $\varGamma _0(N)$, especially $S_{r}^{!,\infty }(\varGamma _0(N))$, the space of weakly holomorphic cusp forms which are holomorphic at every cusp different from $\infty$. Then, we recall their geometric interpretation in terms of the moduli stack ${\mathcal {Y}}_0(N)$, and the *q*-expansion principle. A key result is Lemma 4, which will allow us to control the field of definition of certain Fourier coefficients.

We then proceed to an explicit description of the objects $H_{\mathrm {cusp}}^1({\mathcal {Y}}_0(N),V_k)$ of ${\mathcal {H}}$ in Sects. 4 and 5. This is well known to experts, but we could not find a reference with all the properties, in the particular setting and degree of generality we need, spelled out. After describing the cuspidal de Rham cohomology group $H_{\mathrm {dR},\mathrm {cusp}}^1({\mathcal {Y}}_0(N),{\mathcal {V}}_k)$ (see Corollary 2), we show how to express it as the de Rham realisation of an object in ${\mathcal {H}}$ (see Theorem 5); in particular, it is endowed with a single-valued involution.

In Sect. 6, we ‘compute’ the single-valued involution of $H_{\mathrm {cusp}}^1({\mathcal {Y}}_0(N),V_k)$ in terms of harmonic lifts of modular forms in Theorem 7. Using a theorem of Bringmann and Ono, this allows us to understand the action of the single-valued involution on the cohomology classes defined by Poincaré series (Proposition 7).

Finally, in Sects. 7, 8 and 9, we employ the previous results to prove our main theorems relating the single-valued periods of $H_{\mathrm {cusp}}^1({\mathcal {Y}}_0(N),V_k)$ and the Fourier coefficients at infinity of the Poincaré series $P_{m,k+2,N}$; see Proposition 8, Theorems 8 and 9.

### Terminology and notation

Our notations for modular forms are standard. The Poincaré upper half-plane is denoted by ${\mathbb {H}} = \{\tau \in {\mathbb {C}} \mid \mathfrak {I}\tau >0\}$, and the left action of $\mathrm {SL}_2({\mathbb {R}})$ on ${\mathbb {H}}\cup {\mathbb {P}}^1({\mathbb {R}})$ by

$$\begin{aligned} g\cdot \tau = \frac{a\tau +b}{c\tau +d}\text {, }\qquad \text { where } g = \left( \begin{array}{cc}a &{} b \\ c &{} d\end{array}\right) \text {.} \end{aligned}$$

If $f: {\mathbb {H}} \rightarrow {\mathbb {C}}$ is a function, $g \in \mathrm {SL}_2({\mathbb {R}})$, and *r* is an integer, we set $j(g,\tau )=c\tau +d$, and we denote

$$\begin{aligned} f|_{g,r}: {\mathbb {H}} \rightarrow {\mathbb {C}}\text {, }\qquad \tau \mapsto j(g,\tau )^{-r}f(\tau )\text {.} \end{aligned}$$

If $N\ge 1$ is an integer, the Hecke congruence subgroup of level *N* is the subgroup of $\mathrm {SL}_2({\mathbb {Z}})$ defined by

$$\begin{aligned} \varGamma _0(N) = \left\{ \left( \begin{array}{cc} a &{} b \\ c &{} d\end{array} \right) \in {\mathrm {SL}}_2({\mathbb {Z}})\text { ; } c \equiv 0 \mod N\right\} \text {.} \end{aligned}$$

The set of cusps of $\varGamma _0(N)$ is the quotient $\varGamma _0(N)\backslash {\mathbb {P}}^1({\mathbb {Q}})$. The stabiliser of a cusp *p* is denoted by $\varGamma _0(N)_p$. For $$g = \left( {\begin{matrix}a &{} b \\ c &{} d\end{matrix}}\right) \in \mathrm {SL}_2({\mathbb {Z}})$$, the cusp determined by *g* is the class of (*a* : *c*) in $\varGamma _0(N)\backslash {\mathbb {P}}^1({\mathbb {Q}})$.

We denote

$$\begin{aligned} D = \frac{1}{2\pi i}\frac{d}{d\tau } = q\frac{d}{dq}\text {,} \end{aligned}$$

where $$q= e^{2\pi i \tau }$$.

## The basic formalism of single-valued periods

Let *K* be a subfield of ${\mathbb {R}}$, and ${\mathcal {H}}(K)$ the ‘category of realisations’ considered in [7] (see also [16, 17]). Its objects are given by triples

$$\begin{aligned} H = ((H_{\mathrm {B}}, W^{\mathrm {B}},F_{\infty }),(H_{\mathrm {dR}},W^{\mathrm {dR}},F_{\mathrm {dR}}),\mathrm {comp})\text {,} \end{aligned}$$

where

- $H_{\mathrm {B}}$ is a finite-dimensional ${\mathbb {Q}}$-vector space with an increasing (weight) filtration $W^{\mathrm {B}}$ and an involution $F_{\infty }: H_{\mathrm {B}} \rightarrow H_{\mathrm {B}}$ (‘real Frobenius’),
- $H_{\mathrm {dR}}$ is a finite-dimensional *K*-vector space with an increasing (weight) filtration $W^{\mathrm {dR}}$ and a decreasing (Hodge) filtration $F_{\mathrm {dR}}$, and
- $\mathrm {comp}: H_{\mathrm {dR}}\otimes _{K} {\mathbb {C}} {\mathop {\rightarrow }\limits ^{\sim }} H_{\mathrm {B}}\otimes _{{\mathbb {Q}}}{\mathbb {C}}$ is a ${\mathbb {C}}$-linear isomorphism

such that

- $$\mathrm {comp}(W^{\mathrm {dR}}\otimes _{K}{\mathbb {C}}) = W^{\mathrm {B}}\otimes _{{\mathbb {Q}}}{\mathbb {C}}$$,
- $(H_B, W^{\mathrm {B}}, \mathrm {comp}(F_{\mathrm {dR}}))$ is a ${\mathbb {Q}}$-mixed Hodge structure ([15] 2.3.8), and
- the diagram of ${\mathbb {C}}$-vector spaces 
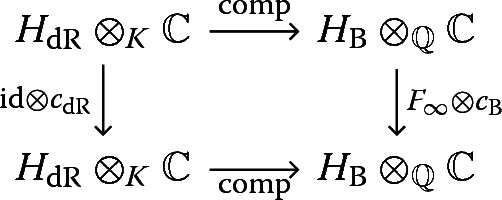
 commutes, where $c_{\mathrm {B}}$ and $c_{\mathrm {dR}}$ denote the action of complex conjugation on coefficients.

A morphism $\varphi : H \rightarrow H'$ is a pair of maps $\varphi _{\mathrm {B}}: H_{\mathrm {B}} \rightarrow H'_{\mathrm {B}}$ (${\mathbb {Q}}$-linear), $\varphi _{\mathrm {dR}}: H_{\mathrm {dR}} \rightarrow H'_{\mathrm {dR}}$ (*K*-linear), ‘preserving’ all of the above structures; in particular, $\varphi _B$ is a morphism of ${\mathbb {Q}}$-mixed Hodge structures $(H_{\mathrm {B}},W^{\mathrm {B}},\mathrm {comp}(F_{\mathrm {dR}})) \rightarrow (H'_{\mathrm {B}},{W^{\mathrm {B}}}', \mathrm {comp}(F_{\mathrm {dR}}'))$.

### Example 3

**(Tate objects)** For every $n \in {\mathbb {Z}}$, we define an object ${\mathbb {Q}}(-n)$ of ${\mathcal {H}}(K)$ as follows: ${\mathbb {Q}}(-n)_{\mathrm {B}} = {\mathbb {Q}}$, $W^{\mathrm {B}}_{2n} = {\mathbb {Q}}(-n)_{\mathrm {B}}$, $W^{\mathrm {B}}_{2n-1} = 0$, $F_{\infty }=(-1)^n\mathrm {id}$, ${\mathbb {Q}}(-n)_{\mathrm {dR}} = K$, $W^{\mathrm {dR}}_{2n} = {\mathbb {Q}}(-n)_{\mathrm {dR}}$, $W^{\mathrm {dR}}_{2n-1} = 0$, $F_{\mathrm {dR}}^n = {\mathbb {Q}}(-n)_{\mathrm {dR}}$, $F_{\mathrm {dR}}^{n+1}=0$, and $\mathrm {comp}: z\mapsto (2\pi i)^nz$.

The commutativity of the diagram (7) implies that the ${\mathbb {C}}$-linear involution $\mathrm {comp}^{-1}\circ (F_{\infty }\otimes \mathrm {id}) \circ \mathrm {comp}$ of $H_{\mathrm {dR}}\otimes _{K}{\mathbb {C}}$ commutes with $\mathrm {id}\otimes c_{\mathrm {dR}}$, so that there exists a unique ${\mathbb {R}}$-linear involution, the *single-valued involution*

$$\begin{aligned} \mathrm {sv}: H_{\mathrm {dR}}\otimes _{K}{\mathbb {R}} \rightarrow H_{\mathrm {dR}}\otimes _{K}{\mathbb {R}}\text {,} \end{aligned}$$

whose ${\mathbb {C}}$-linear extension to $H_{\mathrm {dR}}\otimes _{K}{\mathbb {C}} = (H_{\mathrm {dR}}\otimes _{K}{\mathbb {R}})\otimes _{{\mathbb {R}}}{\mathbb {C}}$ is

8 $$\begin{aligned} \mathrm {sv}\otimes \mathrm {id}= \mathrm {comp}^{-1}\circ (F_{\infty }\otimes \mathrm {id}) \circ \mathrm {comp}\text {.} \end{aligned}$$

Note that a morphism $\varphi : H \rightarrow H'$ in ${\mathcal {H}}(K)$ commutes with the single-valued involutions:

9 $$\begin{aligned} \mathrm {sv}\circ (\varphi _{\mathrm {dR}}\otimes \mathrm {id}) = (\varphi _{\mathrm {dR}}\otimes \mathrm {id})\circ \mathrm {sv}\text {,} \end{aligned}$$

where $\varphi _{\mathrm {dR}}\otimes \mathrm {id}: H_{\mathrm {dR}}\otimes _{K}{\mathbb {R}} \rightarrow H_{\mathrm {dR}}'\otimes _{K}{\mathbb {R}}$ is the ${\mathbb {R}}$-linear extension of $\varphi _{\mathrm {dR}}$.

We can express the action of the Betti complex conjugation $\mathrm {id}\otimes c_{\mathrm {B}}$ on the de Rham side in terms of the single-valued involution as follows.

### Lemma 1

For any object *H* of ${\mathcal {H}}(K)$, we have

$$\begin{aligned} \mathrm {sv}\otimes c_{\mathrm {dR}} = \mathrm {comp}^{-1}\circ (\mathrm {id}\otimes c_{\mathrm {B}}) \circ \mathrm {comp}\end{aligned}$$

on $$H_{\mathrm {dR}}\otimes _{K}{\mathbb {C}}$$.

### Proof

We have:

$$\begin{aligned} \text {sv} \otimes c_{\mathrm {dR}}&= (\text {sv} \otimes \text {id})\circ (\text {id} \otimes c_{\mathrm {dR}})&\\&= \text {comp}^{-1}\circ (F_{\infty }\otimes \text {id}) \circ \text {comp}\circ (\text {id} \otimes c_{\mathrm {dR}})&\text {by definition of }\text {sv} { (8)}\\&= \mathrm {comp}^{-1} \circ (F_{\infty }\otimes \mathrm {id}) \circ (F_{\infty }\otimes c_{\mathrm {B}}) \circ \mathrm {comp}&\text {by the commutativity of }(7)\\&= \mathrm {comp}^{-1}\circ (\mathrm {id}\otimes c_{\mathrm {B}}) \circ \mathrm {comp}&\text {since }F_{\infty }\text { is an involution} \end{aligned}$$

$$\square $$

### Definition 1

A *single-valued period* of an object *H* of ${\mathcal {H}}(K)$ is any real number of the form

$$\begin{aligned} \langle \varphi , \mathrm {sv}(\omega )\rangle \in {\mathbb {R}} \end{aligned}$$

where $\omega \in H_{\mathrm {dR}}$, $\varphi \in H_{\mathrm {dR}}^{\vee }:=\mathrm {Hom}_K(H_{\mathrm {dR}},K)$, and $\langle \ , \ \rangle$ denotes the natural duality pairing.

If *r* denotes the dimension of the *K*-vector space $H_{\mathrm {dR}}$, and

$$\begin{aligned} b_{\mathrm {dR}}: K^{\oplus r} {\mathop {\rightarrow }\limits ^{\sim }} H_{\mathrm {dR}} \end{aligned}$$

is a *K*-basis of $H_{\mathrm {dR}}$, then there exists a unique $S \in \mathrm {GL}_r({\mathbb {R}})$, the matrix of $\mathrm {sv}$ in the basis $b_{\mathrm {dR}}$, such that

$$\begin{aligned} \mathrm {sv}\circ b_{\mathrm {dR}} = b_{\mathrm {dR}} \circ S\text {.} \end{aligned}$$

The coefficients of *S* are single-valued periods of *H* and generate the *K*-linear span of all single-valued periods of *H*. Since $\mathrm {sv}$ is an involution, single-valued periods always satisfy the relations

$$\begin{aligned} S^2 = \mathrm {id}\qquad \text { and }\qquad \text {Tr } S = \text {Tr } F_{\infty }\text {.} \end{aligned}$$

Single-valued periods may also satisfy other relations:

### Example 4

An endomorphism $\varphi$ of *H* in ${\mathcal {H}}(K)$ induces a *K*-linear relation between its single-valued periods. Keeping the above notation, let $M \in \mathrm {GL}_r(K)$ be the matrix of $\varphi _{\mathrm {dR}}$ in the basis $b_{\mathrm {dR}}$, so that $\varphi _{\mathrm {dR}}\circ b_{\mathrm {dR}} = b_{\mathrm {dR}}\circ M$. By (9), and by definition of *M* and *S*, we get

$$\begin{aligned} MS = SM\text {.} \end{aligned}$$

We remark that, if *K* is a real number field and $L\subset {\mathbb {C}}$ is a finite extension of *K*, then we can also define a category of realisations ${\mathcal {H}}(L)$ (see [7] 2.1.2), and we get similar linear relations over *L*, with $M \in \mathrm {GL}_r(L)$, if $\varphi$ is now an endomorphism of $H\otimes _K L$ in ${\mathcal {H}}(L)$.

Let us recall how the notion of single-valued periods connects with the notion of periods (cf. [7] 2.4). The *periods* of an object *H* of ${\mathcal {H}}(K)$ are complex numbers of the form

$$\begin{aligned} \langle \sigma , \mathrm {comp}(\omega ) \rangle \end{aligned}$$

where $\omega \in H_{\mathrm {dR}}$ and $\sigma \in H_B^{\vee } :=\mathrm {Hom}_{{\mathbb {Q}}}(H_{\mathrm {B}},{\mathbb {Q}})$. We can write single-valued periods in terms of periods as follows. If $b_{\mathrm {B}}: {\mathbb {Q}}^{\oplus r} {\mathop {\rightarrow }\limits ^{\sim }} H_{\mathrm {B}}$ is a ${\mathbb {Q}}$-basis of $H_{\mathrm {B}}$, then the *period matrix* of $\mathrm {comp}$ with respect to $b_{\mathrm {dR}}$ and $b_{\mathrm {B}}$ is the unique matrix $P \in \mathrm {GL}_r({\mathbb {C}})$ satisfying

$$\begin{aligned} \mathrm {comp}\circ b_{\mathrm {dR}} = b_{\mathrm {B}} \circ P\text {.} \end{aligned}$$

The coefficients of *P* generate the *K*-linear span of all the periods of *H*. Using Lemma 1 and the definitions of *S* and *P*, we get

$$\begin{aligned} S = {\overline{P}}^{-1}P = P^{-1}{\overline{P}}\text {.} \end{aligned}$$

Further, if we let $R \in \mathrm {GL}_{r}({\mathbb {Q}})$ be such that $F_{\infty }\circ b_{\mathrm {B}} = b_{\mathrm {B}} \circ R$, then ${\overline{P}} = RP$, and we get

$$\begin{aligned} S = P^{-1}RP\text {.} \end{aligned}$$

### Remark 2

Beware that a period of an object *H* of ${\mathcal {H}}(K)$ as defined above is only a period in the sense of Kontsevich and Zagier [23] if *K* is a number field and *H* comes from a mixed motive over *K*, e.g. $H=H^i(X)$ for some *K*-algebraic variety *X*.

## Single-valued periods in the presence of a polarisation

Let *H* be an object of ${\mathcal {H}}(K)$ such that $(H_{\mathrm {B}},W^{\mathrm {B}},\mathrm {comp}(F_{\mathrm {dR}}))$ is a pure ${\mathbb {Q}}$-Hodge structure of weight $n \in {\mathbb {Z}}$. This means that $W^{\mathrm {B}}_n = H_{\mathrm {B}}$, $W^{\mathrm {B}}_{n-1}=0$, and

$$\begin{aligned} H_{\mathrm {B}}\otimes _{{\mathbb {Q}}}{\mathbb {C}} = \bigoplus _{p+q=n} H^{p,q} \end{aligned}$$

where

$$\begin{aligned} H^{p,q} := \mathrm {comp}(F_{\mathrm {dR}}^p) \cap (\mathrm {id}\otimes c_{\mathrm {B}})(\mathrm {comp}(F_{\mathrm {dR}}^q))\text {.} \end{aligned}$$

We say that *H* is *pure of weight n*; the *Hodge type* of *H* is the set $\{(p,q) \in {\mathbb {Z}}^2 \mid H^{p,q}\ne 0\}$.

### Definition 2

A *polarisation* of a pure object *H* of ${\mathcal {H}}(K)$ of weight *n* is a morphism

$$\begin{aligned} \langle \ , \ \rangle : H\otimes H \rightarrow {\mathbb {Q}}(-n) \end{aligned}$$

in ${\mathcal {H}}(K)$ inducing a polarisation on the pure Hodge structure $(H_{\mathrm {B}},\mathrm {comp}(F_{\mathrm {dR}}))$ ([15] 2.1.15). This means that the ${\mathbb {C}}$-linear extension of $\langle \ , \ \rangle _{\mathrm {B}}$ to $H_{\mathrm {B}}\otimes _{{\mathbb {Q}}} {\mathbb {C}}$ is $(-1)^n$-symmetric and satisfies the classical ‘Hodge–Riemann relations’:

1. $$\langle H^{p,q},H^{p',q'}\rangle _{\mathrm {B}} = 0$$ if $$(p,q)\ne (q',p')$$
2. $$i^{p-q}\langle \alpha ,(\mathrm {id}\otimes c_{\mathrm {B}})(\alpha )\rangle _{\mathrm {B}} >0$$ for every $$\alpha \in H^{p,q}\setminus \{0\}$$.

It follows from the commutativity of 
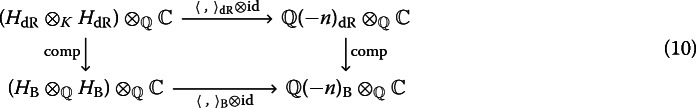
 and from Example 3 that

11 $$\begin{aligned} (2\pi i)^n\langle \omega , \eta \rangle _{\mathrm {dR}} = \langle \mathrm {comp}(\omega ),\mathrm {comp}(\eta )\rangle _{\mathrm {B}} \end{aligned}$$

for every $$\omega ,\eta \in H_{\mathrm {dR}}\otimes _{K}{\mathbb {C}}$$.

### Remark 3

The second Hodge–Riemann relation implies that the ${\mathbb {Q}}$-bilinear pairing $\langle \ , \ \rangle _{\mathrm {B}}$, and hence also the *K*-bilinear pairing $\langle \ , \ \rangle _{\mathrm {dR}}$ by (11), is non-degenerate.

The de Rham pairing $\langle \ , \ \rangle _{\mathrm {dR}}$ is compatible with the single-valued involution as follows.

### Lemma 2

For every $\omega ,\eta \in H_{\mathrm {dR}}\otimes _{K}{\mathbb {C}}$, we have

$$\begin{aligned} \langle (\mathrm {sv}\otimes c_{\mathrm {dR}})(\omega ), (\mathrm {sv}\otimes c_{\mathrm {dR}})(\eta )\rangle _{\mathrm {dR}} = (-1)^n\overline{\langle \omega ,\eta \rangle }_{\mathrm {dR}}\text {.} \end{aligned}$$

### Proof

Since the de Rham pairing is defined over $K\subset {\mathbb {R}}$, it suffices to prove the above formula for $\omega ,\eta \in H_{\mathrm {dR}}$. In this case, we have $(\mathrm {sv}\otimes c_{\mathrm {dR}})(\omega ) = \mathrm {sv}(\omega )$ and similarly for $\eta$. The equality $\langle \mathrm {sv}(\omega ),\mathrm {sv}(\eta )\rangle _{\mathrm {dR}} = (-1)^n\langle \omega ,\eta \rangle _{\mathrm {dR}}$ then follows from (9) and from the fact that the single-valued involution of ${\mathbb {Q}}(-n)$ is $(-1)^n$ (Example 3).

Alternatively, we may apply Lemma 1 together with the formula (11), and use that $\langle (\mathrm {id}\otimes c_{\mathrm {B}})(\alpha ), (\mathrm {id}\otimes c_{\mathrm {B}})(\beta ) \rangle _{\mathrm {B}} = \overline{\langle \alpha ,\beta \rangle }_{\mathrm {B}}$ for every $\alpha ,\beta \in H_{\mathrm {B}}\otimes _{{\mathbb {Q}}}{\mathbb {C}}$. $\square$

The presence of a polarisation imposes additional algebraic relations on single-valued periods. For future reference, we spell out these relations in the particular case where *H* is pure of Hodge type $\{(n,0),(0,n)\}$ for some $n\in {\mathbb {Z}}$.

In this case, let $d :=\dim F_{\mathrm {dR}}^n$, so that $\dim H_{\mathrm {dR}} = 2d$. Consider a *K*-basis

12 $$\begin{aligned} b_{\mathrm {dR}} = (\omega _1,\ldots ,\omega _d,\eta _1,\ldots ,\eta _d) \end{aligned}$$

of $H_{\mathrm {dR}}$ such that

- $(\omega _1,\ldots ,\omega _d)$ is a *K*-basis of $F_{\mathrm {dR}}^n$;
- $\langle \omega _i, \eta _j \rangle _{\mathrm {dR}} = \delta _{ij}$ and $\langle \eta _i,\eta _j \rangle _{\mathrm {dR}} = 0$ for every $1\le i,j\le d$.

Note that $\langle \omega _i, \omega _j \rangle _{\mathrm {dR}}=0$ because $F_{\mathrm {dR}}^n$ is isotropic for $\langle \ , \ \rangle _{\mathrm {dR}}$ by the first Hodge–Riemann relation. Such bases always exist by a simple variation of the Gram–Schmidt process (cf. Remark 3).

Let us write the matrix *S* of the single-valued involution $\mathrm {sv}: H_{\mathrm {dR}}\otimes _{K}{\mathbb {R}} \rightarrow H_{\mathrm {dR}}\otimes _{K}{\mathbb {R}}$ in the basis $b_{\mathrm {dR}}$ in block form:

$$\begin{aligned} S= \left( \begin{array}{cc} A &{} B \\ C &{} D \end{array}\right) \in {\mathrm {GL}}_{2d}({\mathbb {R}})\text {,} \end{aligned}$$

where $$A,B,C,D \in M_{d\times d}({\mathbb {R}})$$.

### Proposition 2

Let *H* be a polarisable pure object of ${\mathcal {H}}(K)$, pure of Hodge type $\{(n,0),(0,n)\}$ for some $n\in {\mathbb {Z}}$. With the above notation, we have $C \in \mathrm {GL}_d({\mathbb {R}})$, and

$$\begin{aligned} B = (1-A^2)C^{-1}\text {, }\ \ \ D=(-1)^{n}A^t\text {,}\ \ \ CA = (-1)^{n+1}A^tC\text {, } \ \ \ C = C^t\text {.} \end{aligned}$$

In particular, the field *K*(*S*) generated by all of the coefficients of *S* is equal to the field *K*(*A*, *C*), generated by the coefficients of *A* and *C*.

In coordinate-free parlance, the field of rationality of the single-valued involution $\mathrm {sv}: H_{\mathrm {dR}}\otimes _{K} {\mathbb {R}} \rightarrow H_{\mathrm {dR}}\otimes _{K} {\mathbb {R}}$ coincides with the field of rationality of its restriction $\mathrm {sv}|_{F_{\mathrm {dR}}^n\otimes _K{\mathbb {R}}}: F_{\mathrm {dR}}^n\otimes _K{\mathbb {R}} \rightarrow H_{\mathrm {dR}}\otimes _{K} {\mathbb {R}}$.

### Proof

Since $\omega _i$ are defined over $K\subset {\mathbb {R}}$, we have $(\mathrm {sv}\otimes c_{\mathrm {dR}})(\omega _i) = \mathrm {sv}(\omega _i)$. It follows from Lemma 1 and from the Hodge decomposition $H_{\mathrm {B}}\otimes _{{\mathbb {Q}}}{\mathbb {C}} = H^{n,0} \oplus H^{0,n}$ that

$$\begin{aligned} (\omega _1,\ldots ,\omega _d,\mathrm {sv}(\omega _1),\ldots ,\mathrm {sv}(\omega _d)) = (\omega _1,\ldots ,\omega _d,\eta _1,\ldots ,\eta _d) \cdot \left( \begin{array}{cc} 1 &{} A \\ 0 &{} C \end{array}\right) \end{aligned}$$

is a basis of $H_{\mathrm {dR}}\otimes _K{\mathbb {R}}$; this proves that $C \in \mathrm {GL}_{d}({\mathbb {R}})$.

It follows from the definition of the basis $b_{\mathrm {dR}}$, and from Lemma 2, that

$$\begin{aligned} S^t \left( \begin{array}{cc} 0 &{} (-1)^n \\ 1 &{} 0 \end{array}\right) S = (-1)^n \left( \begin{array}{cc} 0 &{} (-1)^n \\ 1 &{} 0 \end{array}\right) \end{aligned}$$

or, equivalently, using that $S^{-1}=S$,

$$\begin{aligned} \left( \begin{array}{cc} C^t &{} (-1)^nA^t \\ D^t &{} (-1)^nB^t \end{array}\right) = \left( \begin{array}{cc} C &{} D \\ (-1)^nA &{} (-1)^nB \end{array}\right) \end{aligned}$$

This proves the relations $D=(-1)^nA^t$ and $C=C^t$.

Finally, the equation $S^2=1$ gives

$$\begin{aligned} S^2 = \left( \begin{array}{cc} A^2 +BC &{} AB+BD \\ CA +DC &{} CB+D^2 \end{array}\right) = \left( \begin{array}{cc} 1 &{} 0 \\ 0 &{} 1 \end{array}\right) \end{aligned}$$

so that $B= (1-A^2)C^{-1}$, and $CA = -DC = (-1)^{n+1}A^tC$. $\square$

### Remark 4

With the above notation, set $R :=AC^{-1}$. Then, $R^t=(-1)^{n+1}R$, $C^t=C$, and we have

$$\begin{aligned} S = \left( \begin{array}{cc} RC &{} C^{-1} - RCR \\ C &{} -CR \end{array}\right) \in {\mathrm {GL}}_{2d}({\mathbb {R}})\text {.} \end{aligned}$$

We finish this section with an alternative way of encoding the single-valued period matrix, in terms of the Hermitian form defined by the polarisation.

### Lemma 3

The ${\mathbb {R}}$-bilinear form on $H_{\mathrm {dR}}\otimes _{K}{\mathbb {C}}$ with values in ${\mathbb {C}}$ defined by

$$\begin{aligned} ( \omega ,\eta )_{\mathrm {dR}} :=(-1)^n\langle \omega , (\mathrm {sv}\otimes c_{\mathrm {dR}})(\eta )\rangle _{\mathrm {dR}} \end{aligned}$$

is Hermitian. Moreover, the restriction of $( \ , \ )_{\mathrm {dR}}$ to $F_{\mathrm {dR}}^n\otimes _{K}{\mathbb {C}}$ is positive definite.

### Proof

That $( \ , \ )_{\mathrm {dR}}$ is a Hermitian form (i.e. $( z\omega , w\eta )_{\mathrm {dR}} = z{\overline{w}}(\omega ,\eta )_{\mathrm {dR}}$ for $z,w \in {\mathbb {C}}$) follows from formula (11) and from Lemma 1. The positive definiteness on $F_{\mathrm {dR}}^n\otimes _{K}{\mathbb {C}}$ is a consequence of the second Hodge–Riemann relation. $\square$

Consider a de Rham basis $b_{\mathrm {dR}} = (\omega _1,\ldots ,\omega _d,\eta _1,\ldots ,\eta _d)$ as above. Then

13 $$\begin{aligned} \begin{aligned} (\omega _i,\omega _j)_{\mathrm {dR}}&= (-1)^n\langle \omega _i, \mathrm {sv}(\omega _j)\rangle _{\mathrm {dR}}\\&=(-1)^n\sum _{k=1}^d\left( A_{kj}\langle \omega _i,\omega _k\rangle _{\mathrm {dR}} + C_{kj}\langle \omega _i,\eta _k \rangle _{\mathrm {dR}}\right) \\&= (-1)^nC_{ij}\text {,} \end{aligned} \end{aligned}$$

and, similarly,

14 $$\begin{aligned} (\omega _i,\eta _j)_{\mathrm {dR}} = (-1)^nD_{ij}\text {, }\ \ \ (\eta _i,\omega _j)_{\mathrm {dR}} = A_{ij}\text {, } \ \ \ (\eta _i,\eta _j)_{\mathrm {dR}} = B_{ij}\text {.} \end{aligned}$$

Note that we proved in Proposition 2 that *C* is invertible; using the above formulas, we see that this is equivalent to the positive definiteness of $( \ , \ )_{\mathrm {dR}}$ over $F_{\mathrm {dR}}^n\otimes _K{\mathbb {C}}$ (Lemma 3).

## Spaces of weakly holomorphic modular forms

Let *r* and $N\ge 1$ be integers.

### Definition 3

A *weakly holomorphic modular form* of weight *r* and level $\varGamma _0(N)$ is a holomorphic function $f: {\mathbb {H}} \rightarrow {\mathbb {C}}$ which is modular of weight *r* for $\varGamma _0(N)$:

$$\begin{aligned} f|_{\gamma ,r} = f \end{aligned}$$

for every $\gamma \in \varGamma _0(N)$, and meromorphic at all cusps: for every $g \in \mathrm {SL}_2({\mathbb {Z}})$, there exists $\rho >0$ such that

$$\begin{aligned} f|_{g,r}= O(e^{\rho \mathfrak {I}\tau }) \end{aligned}$$

as $$\mathfrak {I}\tau \rightarrow +\infty $$.

The ${\mathbb {C}}$-vector space of weakly holomorphic modular forms of weight *r* and level $\varGamma _0(N)$ is denoted by $M^!_r(\varGamma _0(N))$. Note that $M_r^!(\varGamma _0(N))=0$ for every odd *r*, since $$\bigl ( {\begin{matrix}-1 &{} 0\\ 0 &{} -1\end{matrix}}\bigr ) \in \varGamma _0(N)$$.

Every $f \in M_r^!(\varGamma _0(N))$ admits a *Fourier expansion at infinity*, which we denote by

$$\begin{aligned} f = \sum _{n \gg -\infty }a_n(f)q^n \end{aligned}$$

where $q = e^{2\pi i \tau }$, and $a_n(f) \in {\mathbb {C}}$. For every $n \in {\mathbb {Z}}$, we can regard

$$\begin{aligned} a_n : M_r^!(\varGamma _0(N)) \rightarrow {\mathbb {C}}\text {, }\qquad f \mapsto a_n(f) \end{aligned}$$

as a linear functional on $M_r^!(\varGamma _0(N))$. If *K* is any subfield of ${\mathbb {C}}$, we denote by

$$\begin{aligned} M_r^!(\varGamma _0(N);K) \end{aligned}$$

the *K*-subspace of $M_r^!(\varGamma _0(N))$ consisting of those *f* such that $a_n(f)\in K$ for every $n \in {\mathbb {Z}}$.

### Definition 4

For $f \in M_r^!(\varGamma _0(N);K)$, the (extended) *principal part of f at the cusp at infinity* is defined by the finite sum ${\mathcal {P}}_f = \sum _{n\le 0}a_n(f)q^n \in K[q^{-1}]$.

There are also Fourier expansions at other cusps. Let

$$\begin{aligned} g = \left( \begin{array}{cc} a &{} b \\ c &{} d \end{array}\right) \in {\mathrm {SL}}_2({\mathbb {Z}})\text {.} \end{aligned}$$

If *w* denotes the smallest positive integer such that $$\bigl ( {\begin{matrix}1 &{} w\\ 0 &{} 1\end{matrix}}\bigr ) \in g^{-1}\varGamma _0(N)g$$ (the ‘*width* of the cusp determined by *g*’), then we can write

$$\begin{aligned} f|_{g,r} = \sum _{n\gg -\infty }a_{n,g}(f) q^{\frac{n}{w}} \end{aligned}$$

for some $a_{n,g}(f) \in {\mathbb {C}}$. This is a ‘Fourier expansion of *f* at the cusp $[(a:c)] \in \varGamma _0(N)\backslash {\mathbb {P}}^1({\mathbb {Q}})$’, but it really depends on *g*: for $j \in {\mathbb {Z}}$, we have

$$\begin{aligned} a_{n,gT^j} = e^{2\pi i \frac{j}{w}}a_{n,g}\text {,} \end{aligned}$$

where $$T= \left( {\begin{matrix}1 &{} 1 \\ 0 &{} 1\end{matrix}}\right) $$. Note, however, that the vanishing of the *n*th Fourier coefficient at a given cusp is a well defined property.

### Definition 5

A *weakly holomorphic cusp form* of weight *r* and level $\varGamma _0(N)$ is a weakly holomorphic modular form $f \in M_r^!(\varGamma _0(N))$ such that $a_{0,g}(f)=0$ for every $g \in \mathrm {SL}_2({\mathbb {Z}})$.

We denote the subspace of weakly holomorphic cusp forms of weight *r* and level $\varGamma _0(N)$ by $S_r^!(\varGamma _0(N)) \subset M_r^!(\varGamma _0(N))$. Similarly, for every subfield $K\subset {\mathbb {C}}$, we set

$$\begin{aligned} S_r^!(\varGamma _0(N);K) :=S_r^!(\varGamma _0(N))\cap M_r^!(\varGamma _0(N);K)\text {.} \end{aligned}$$

Note that the space $M_r(\varGamma _0(N))$ (resp. $S_r(\varGamma _0(N))$) of modular forms (resp. cusp forms) of weight *r* and level $\varGamma _0(N)$ is the subspace of $M_r^!(\varGamma _0(N))$ consisting of those *f* such that $a_{n,g}(f)=0$ for every $n<0$ (resp. $n\le 0$) and every $g \in \mathrm {SL}_2({\mathbb {Z}})$. For a subfield *K* of ${\mathbb {C}}$, we also set

$$\begin{aligned} M_r(\varGamma _0(N);K) :=M_r(\varGamma _0(N))\cap M_r^!(\varGamma _0(N);K) \end{aligned}$$

and

$$\begin{aligned} S_r(\varGamma _0(N);K) :=S_r(\varGamma _0(N))\cap S_r^!(\varGamma _0(N);K) \text {.} \end{aligned}$$

### Remark 5

Recall that we always have $M_r(\varGamma _0(N))=0$ for $r<0$, although $M_r^!(\varGamma _0(N))$ can be non-trivial. This follows from the ‘valence formula’ (see [12] 5.6.1): for every $f \in M_{r}^{!}(\varGamma _0(N))$,

$$\begin{aligned} \frac{1}{[\mathrm {SL}_2({\mathbb {Z}}) : \varGamma _0(N)]}\sum _{p \in \varGamma _0(N)\backslash {\mathbb {H}} \cup {\mathbb {P}}^1({\mathbb {Q}})}\frac{\mathrm {ord}_{p}(f)}{e_p} = \frac{r}{12} \end{aligned}$$

where $e_{p}=2$ (resp. $e_{p}=3$) if $p= \varGamma _0(N)\cdot i$ (resp. $p= \varGamma _0(N)\cdot e^{\frac{2\pi i}{3} }$), and $e_{p}=1$ otherwise.

We shall also consider weakly holomorphic modular forms which are holomorphic at all *finite* cusps. Namely, we define a subspace $M_r^{!,\infty }(\varGamma _0(N))$ of $M^!_r(\varGamma _0(N))$ consisting of *f* such that $a_{n,g}(f)=0$ for every $n<0$ and $$g = \bigl ( {\begin{matrix}a &{} b\\ c &{} d\end{matrix}}\bigr ) \in \mathrm {SL}_2({\mathbb {Z}})$$ with $c\not \equiv 0 \pmod N$. In other words, $M_r^{!,\infty }(\varGamma _0(N))$ is the space of weakly holomorphic modular forms with no pole at any cusp different from $\infty$, but which are allowed to have poles at $\infty$. We also define $S_r^{!,\infty }(\varGamma _0(N)) :=S_r^!(\varGamma _0(N)) \cap M_{r}^{!,\infty }(\varGamma _0(N))$ and, for a subfield *K* of ${\mathbb {C}}$,

$$\begin{aligned} M_{r}^{!,\infty }(\varGamma _0(N);K) :=M_r^{!,\infty }(\varGamma _0(N)) \cap M_r^!(\varGamma _0(N);K) \end{aligned}$$

and

$$\begin{aligned} S_{r}^{!,\infty }(\varGamma _0(N);K) :=S_r^{!,\infty }(\varGamma _0(N)) \cap S_r^!(\varGamma _0(N);K)\text {.} \end{aligned}$$

We then have the following diagram of *K*-vector spaces, where all the arrows are the natural inclusions 
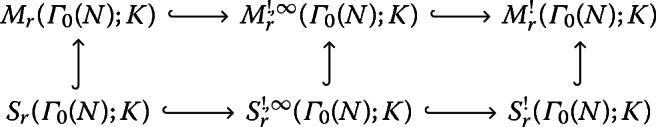
 Note that $M_r(\varGamma _0(N);K)$ (resp. $S_r(\varGamma _0(N);K)$) is the subspace of $M^{!,\infty }_r(\varGamma _0(N);K)$ of weakly holomorphic modular forms with constant (resp. vanishing) principal part at the cusp at infinity.

We conclude this subsection with the example of Poincaré series. Let $\varGamma _{\infty } \le \varGamma _0(N)$ be the stabiliser of $\infty =(1:0) \in {\mathbb {P}}^1({\mathbb {Q}})$. The proofs of the following propositions are well known in weight $k\ge 4$; see, for instance, [12] Chapter 8. For Poincaré series of weight 2, a precise exposition can be found in [30] 5.7.

### Proposition 3

Let $k\ge 2$ and *m* be integers, with *k* even.

1. If $k\ge 4$, then the series
  $$\begin{aligned} P_{m,k,N}(\tau ) = \sum _{\gamma \in \varGamma _{\infty }\backslash \varGamma _0(N)}\frac{e^{2\pi i m \gamma \cdot \tau }}{j(\gamma ,\tau )^k} \end{aligned}$$
  is uniformly absolutely convergent on sets of the form $\{\tau \in {\mathbb {H}} \mid |\mathfrak {R}\tau |\le \alpha \text {, } \mathfrak {I}\tau \ge \beta \}$, for $\alpha ,\beta > 0$, and we have
  $$\begin{aligned} P_{m,k,N} \in S_k^{!,\infty }(\varGamma _0(N);{\mathbb {R}})\text {.} \end{aligned}$$
2. If $k=2$, then the series
  $$\begin{aligned} P_{m,2,N}(\tau ,\epsilon ) = \sum _{\gamma \in \varGamma _{\infty }\backslash \varGamma _0(N)}\frac{e^{2\pi i m \gamma \cdot \tau }}{j(\gamma ,\tau )^2|j(\gamma ,\tau )|^{2\epsilon }} \end{aligned}$$
  is uniformly absolutely convergent on sets of the form $\{(\tau ,\epsilon ) \in {\mathbb {H}} \times {\mathbb {R}} \mid |\mathfrak {R}\tau |\le \alpha \text {, } \mathfrak {I}\tau \ge \beta \text {, }\epsilon \ge \epsilon _0\}$, for $\alpha ,\beta ,\epsilon _0 > 0$. For every $\gamma \in \varGamma _0(N)$, we have
  $$\begin{aligned} P_{m,2,N}(\gamma \cdot \tau ,\epsilon ) = j(\gamma ,\tau )^2|j(\gamma ,\tau )|^{2\epsilon } P_{m,2,N}( \tau ,\epsilon )\text {,} \end{aligned}$$
  and, if $m\ne 0$, then
  $$\begin{aligned} P_{m,2,N}:=\lim _{\epsilon \rightarrow 0+}P_{m,2,N}(\cdot ,\epsilon ) \in S_{2}^{!,\infty }(\varGamma _0(N);{\mathbb {R}})\text {.} \end{aligned}$$

Recall that, for $a,b,c \in {\mathbb {Z}}$, with $c\ge 1$, the Kloosterman sum *K*(*a*, *b*; *c*) is the real algebraic number defined by

$$\begin{aligned} K(a,b;c) = \sum _{x \in ({\mathbb {Z}}/c{\mathbb {Z}})^{\times }}e^{\frac{2\pi i}{m}(ax + bx^{-1})}\text {.} \end{aligned}$$

### Proposition 4

Let $k\ge 2$ and $m\ge 1$ be integers, with *k* even.

1. We have $P_{m,k,N} \in S_k(\varGamma _0(N);{\mathbb {R}})$ and, for every integer $n\ge 1$,
  $$\begin{aligned} a_n(P_{m,k,N}) = \delta _{m,n}+ 2\pi (-1)^{\frac{k}{2}}\left( \frac{n}{m} \right) ^{\frac{k-1}{2}}\sum _{c\ge 1\text {, }N \mid c}\frac{K(m,n;c)}{c}J_{k-1}\left( \frac{4\pi \sqrt{mn}}{c} \right) \end{aligned}$$
  where $J_{k-1}$ is the *J*-Bessel function of order $k-1$ (see [30] (5.3.2)).
2. The principal part of $P_{-m,k,N}$ at the cusp at infinity is $q^{-m}$, and, for every integer $n\ge 1$,
  $$\begin{aligned} a_n(P_{-m,k,N}) = 2\pi (-1)^{\frac{k}{2}} \left( \frac{n}{m} \right) ^{\frac{k-1}{2}}\sum _{c\ge 1\text {, }N\mid c}\frac{K(-m,n;c)}{c}I_{k-1}\left( \frac{4\pi \sqrt{mn}}{c}\right) \end{aligned}$$
  where $I_{k-1}$ is the *I*-Bessel function of order $k-1$ (see [30] (5.3.3)).

## Geometric interpretation; the *q*-expansion principle

We work over the moduli stacks ${\mathcal {Y}}_0(N)$ and ${\mathcal {X}}_0(N)$ over $\mathrm {Spec}\, {\mathbb {Q}}$. By definition, for every ${\mathbb {Q}}$-scheme *S*, the fibre of ${\mathcal {Y}}_0(N)$ at *S* is given by pairs (*E*, *C*), where *E* is an elliptic curve over *S*, and *C* is a cyclic *S*-subgroup scheme of *E* of order *N* (i.e. locally for the fppf topology on *S*, the subgroup *C* admits a generator of order *N*; see [21] 3.4). The ‘compactified’ moduli stack ${\mathcal {X}}_0(N)$ has a similar definition, but now *E* is allowed to be a generalised elliptic curve, as in [18].

One can prove that ${\mathcal {X}}_0(N)$ is a proper smooth Deligne–Mumford stack over $\mathrm {Spec}\, {\mathbb {Q}}$ containing ${\mathcal {Y}}_0(N)$ as an open substack. More precisely, it follows from [18], Thérème 3.4, that ${\mathcal {Y}}_0(N)$ is the complement of a closed substack ${\mathcal {Z}}_N\subset {\mathcal {X}}_0(N)$, the *cuspidal locus of* ${\mathcal {X}}_0(N)$, which is finite étale over $\mathrm {Spec}\, {\mathbb {Q}}$ (therefore representable by a ${\mathbb {Q}}$-scheme).

### Remark 6

The moduli stacks ${\mathcal {Y}}_0(1)$ and ${\mathcal {X}}_0(1)$ are often denoted by ${\mathcal {M}}_{1,1}$ and $\overline{{\mathcal {M}}}_{1,1}$ in the literature.

In the category of complex analytic spaces, let ${\mathbb {E}}$ be the elliptic curve over ${\mathbb {H}}$ whose fibre at $\tau \in {\mathbb {H}}$ is the complex torus

$$\begin{aligned} {\mathbb {E}}_{\tau } = {\mathbb {C}}/({\mathbb {Z}} + \tau {\mathbb {Z}})\text {.} \end{aligned}$$

Then, the map

15 $$\begin{aligned} {\mathbb {H}} \rightarrow {\mathcal {Y}}_0(N)_{{\mathbb {C}}}^{\mathrm {an}}\text {, } \qquad \tau \mapsto ({\mathbb {E}}_{\tau },(\tfrac{1}{N}{\mathbb {Z}} + \tau {\mathbb {Z}})/({\mathbb {Z}} + \tau {\mathbb {Z}})) \end{aligned}$$

induces an isomorphism

$$\begin{aligned} {\mathcal {Y}}_0(N)_{{\mathbb {C}}}^{\mathrm {an}} \cong \varGamma _0(N)\backslash \! \backslash {\mathbb {H}}\text {,} \end{aligned}$$

where the double backslash stands for the ‘orbifold quotient’ (or ‘stacky quotient’) of ${\mathbb {H}}$ by the left action of $\varGamma _0(N)$.

For $g\in \mathrm {SL}_2({\mathbb {Z}})$, and *w* the width of the cusp *p* determined by *g*, a coordinate chart of the ‘compactification’ ${\mathcal {X}}_0(N)_{{\mathbb {C}}}^{\mathrm {an}}$ of ${\mathcal {Y}}_0(N)_{{\mathbb {C}}}^{\mathrm {an}}$ at a neighbourhood of *p* is induced by

16 $$\begin{aligned} \tau \mapsto e^{2\pi i\frac{g^{-1}\cdot \tau }{w}}\text {.} \end{aligned}$$

### Remark 7

The moduli stacks ${\mathcal {Y}}_0(N)$ and ${\mathcal {X}}_0(N)$ are *never* representable by schemes. Indeed, any subgroup of an elliptic curve *E* is preserved by multiplication by $-1$; thus, any object of ${\mathcal {Y}}_0(N)$ admits non-trivial automorphisms. If *p* is an odd prime not dividing *N*, the moduli stack ${\mathcal {Y}}_0(N)_p$ classifying elliptic curves with a cyclic subgroup of order *N* *and* a full level *p* structure is representable by a smooth affine ${\mathbb {Q}}$-scheme *Y* with a $\mathrm {GL}_2({\mathbb {F}}_p)$-action (cf. [18] Théorème 2.7). The natural map $Y\rightarrow {\mathcal {Y}}_0(N)$ is finite étale and induces an isomorphism ${\mathcal {Y}}_0(N) \cong Y/\!/\mathrm {GL}_2({\mathbb {F}}_p)$. Similar considerations also hold for the compactified modular curve ${\mathcal {X}}_0(N)$.

Let ${\mathcal {F}}$ be the *Hodge line bundle* over ${\mathcal {Y}}_0(N)$. By definition, if $\pi : {\mathcal {E}} \rightarrow {\mathcal {Y}}_0(N)$ denotes the universal elliptic curve over ${\mathcal {Y}}_0(N)$, then ${\mathcal {F}} :=\pi _* \varOmega ^{1}_{{\mathcal {E}}/{\mathcal {Y}}_0(N)}$. Concretely, for a *k*-point $y:\mathrm {Spec}\, k \rightarrow {\mathcal {Y}}_0(N)$ corresponding to a pair (*E*, *C*) over a field *k*, the fibre of ${\mathcal {F}}$ at *y* is given by ${\mathcal {F}}(y) = H^0(E,\varOmega ^1_{E/k})$. The Hodge line bundle can be extended to a line bundle $\overline{{\mathcal {F}}}$ over ${\mathcal {X}}_0(N)$ by setting $\overline{{\mathcal {F}}}:={\overline{\pi }}_* \varOmega ^{1}_{\overline{{\mathcal {E}}}/{\mathcal {X}}_0(N)}(\log {\overline{\pi }}^{-1}({\mathcal {Z}}_N))$, where ${\overline{\pi }}: \overline{{\mathcal {E}}} \rightarrow {\mathcal {X}}_0(N)$ denotes the universal generalised elliptic curve over ${\mathcal {X}}_0(N)$.

### Example 5

The pullback of ${\mathcal {F}}^{\mathrm {an}}$ to ${\mathbb {H}}$ via the uniformisation map (15) is trivialised by the global section $\omega \in H^0({\mathbb {H}}, {\mathcal {F}}^{\mathrm {an}})$ whose fibre at $\tau \in {\mathbb {H}}$ is the holomorphic 1-form

$$\begin{aligned} \omega _{\tau } :=2\pi i\, dz \end{aligned}$$

on the complex torus ${\mathbb {E}}_{\tau } = {\mathbb {C}}/({\mathbb {Z}} + \tau {\mathbb {Z}})$. Note that every $\gamma \in \varGamma _0(N)$ defines an automorphism of ${\mathbb {E}}$ by

$$\begin{aligned} \gamma _\tau : {\mathbb {E}}_{\tau } \rightarrow {\mathbb {E}}_{\gamma \cdot \tau }\text {, }\qquad z \mapsto j(\gamma ,\tau )^{-1}z\text {,} \end{aligned}$$

so that it acts on ${\mathcal {F}}^{\mathrm {an}}$ by pullback. Explicitly, $\gamma ^*\omega = j(\gamma ,\tau )^{-1}\omega$.

Let *r* be an integer. If $f \in M^!_{r}(\varGamma _0(N))$, then it follows from the above example that the section $f\omega ^{\otimes r} \in H^0({\mathbb {H}}, ({\mathcal {F}}^{\mathrm {an}})^{\otimes r})$ is $\varGamma _0(N)$-invariant, so that it descends to a global section $\omega _f$ in $H^0({\mathcal {Y}}_0(N)^{\mathrm {an}}_{{\mathbb {C}}}, ({\mathcal {F}}^{\mathrm {an}})^{\otimes r})$.

### Proposition 5

(*q*–expansion principle; cf. [20] 1.6) The section $\omega _f$ constructed above is algebraic, i.e. $\omega _f \in H^0({\mathcal {Y}}_0(N)_{{\mathbb {C}}},{\mathcal {F}}^{\otimes r}) = H^0({\mathcal {Y}}_0(N),{\mathcal {F}}^{\otimes r})\otimes _{{\mathbb {Q}}}{\mathbb {C}}$. Moreover, $f \in M^!_r(\varGamma _0(N);{\mathbb {Q}})$ if and only if $\omega _f \in H^0({\mathcal {Y}}_0(N), {\mathcal {F}}^{\otimes r})$.

### sketch

Let *p* be an odd prime not dividing *N*, so that ${\mathcal {X}}_0(N)_p$ (resp. ${\mathcal {Y}}_0(N)_p$) is representable by a ${\mathbb {Q}}$-scheme *X* (resp. *Y*) (see Remark 7). By the growth condition of *f* at the cusps, $\omega _f$ extends to a *meromorphic* global section of $(\overline{{\mathcal {F}}}^{\mathrm {an}})^{\otimes r}$ over $X_{{\mathbb {C}}}^{\mathrm {an}}$. Since $\overline{{\mathcal {F}}}$ is an algebraic coherent sheaf, and since $X_{{\mathbb {C}}}$ is projective, Serre’s GAGA implies that the pullback of $\omega _f$ to $X_{{\mathbb {C}}}^{\mathrm {an}}$ is given by a *rational* global section of $\overline{{\mathcal {F}}}^{\otimes r}$ over $X_{{\mathbb {C}}}$; since it is regular over $Y_{{\mathbb {C}}}$, it belongs to $H^0(Y_{{\mathbb {C}}}, {\mathcal {F}}^{\otimes r})$. By considering $\mathrm {GL}_{2}({\mathbb {F}}_p)$-invariants, we conclude that $\omega _f \in H^0({\mathcal {Y}}_0(N)_{{\mathbb {C}}},{\mathcal {F}}^{\otimes r})$.

Note that the uniformisation map ${\mathbb {H}} \rightarrow {\mathcal {Y}}_0(N)_{{\mathbb {C}}}^{\mathrm {an}}$ (15) factors through a map ${\mathbb {D}}^* \rightarrow {\mathcal {Y}}_0(N)_{{\mathbb {C}}}^{\mathrm {an}}$ via ${\mathbb {H}} \rightarrow {\mathbb {D}}^*$, $\tau \mapsto q(\tau )=e^{2\pi i \tau }$, where ${\mathbb {D}}^*$ denotes the punctured complex unit disc. The section $\omega$ over ${\mathbb {H}}$ also descends to ${\mathbb {D}}^*$, so that the pullback of $\omega _f$ to ${\mathbb {D}}^*$ is simply $f(q)\omega ^{\otimes r}$, where *f*(*q*) denotes the Fourier expansion of *f* at infinity. We now remark that ${\mathbb {D}}^* \rightarrow {\mathcal {Y}}_0(N)_{{\mathbb {C}}}^{\mathrm {an}}$ ‘descends’ to an algebraic morphism $\mathrm {Spec}\, {\mathbb {Q}}(\!(q)\!) \rightarrow {\mathcal {Y}}_0(N)$, given by the *Tate curve* (see [18] VII.4). From this, we conclude that $\omega _f \in H^0({\mathcal {Y}}_0(N), {\mathcal {F}}^{\otimes r})$ if and only if $f(q) \in {\mathbb {Q}}(\!(q)\!)$. $\square$

### Corollary 1

For every integer *r* and every subfield *K* of ${\mathbb {C}}$, the map

$$\begin{aligned} M_{r}^!(\varGamma _0(N);K) \rightarrow H^0({\mathcal {Y}}_0(N)_K,{\mathcal {F}}^{\otimes r})\text {, }\qquad f \mapsto \omega _f \end{aligned}$$

is an isomorphism of *K*-vector spaces. In particular,

$$\begin{aligned} M_r^!(\varGamma _0(N);K) = M_r^!(\varGamma _0(N);{\mathbb {Q}})\otimes _{{\mathbb {Q}}} K\text {.} \end{aligned}$$

It follows from the explicit description of the coordinates at the cusps of ${\mathcal {X}}_0(N)_{{\mathbb {C}}}^{\mathrm {an}}$ given in (16) that, if $f \in M_r(\varGamma _0(N))$, then $\omega _f$ extends holomorphically to every cusp. Thus,

$$\begin{aligned} M_r(\varGamma _0(N);K) \cong H^0({\mathcal {X}}_0(N)_K,\overline{{\mathcal {F}}}^{\otimes r})\text {,} \end{aligned}$$

for every subfield $K\subset {\mathbb {C}}$. Similarly, $M_r^{!,\infty }(\varGamma _0(N))$ corresponds to those elements of $H^0({\mathcal {Y}}_0(N)_{{\mathbb {C}}},{\mathcal {F}}^{\otimes r})$ which extend holomorphically at every cusp different from $\infty$, and the space $S_r^{!}(\varGamma _0(N))$ is given by the elements of $H^0({\mathcal {Y}}_0(N)_{{\mathbb {C}}},{\mathcal {F}}^{\otimes r})$ with trivial residue at every cusp.

The following technical lemma will be helpful throughout this paper.

### Lemma 4

Let $k\ge 0$ be an even integer, and $f \in M_{-k}^{!,\infty }(\varGamma _0(N))$. If *K* denotes the subfield of ${\mathbb {C}}$ generated by $a_n(f)$ for $n\le 0$, then $f \in M_{-k}^{!,\infty }(\varGamma _0(N);K)$.

In other words, for any weakly holomorphic modular form of negative or zero weight, its field of definition is determined by its principal part.

### Proof

Since there are no nonzero modular forms of negative weight, and the only modular forms in weight 0 are constant (Remark 5)^2^, the ${\mathbb {C}}$-linear map $M_{-k}^{!,\infty }(\varGamma _0(N)) \rightarrow {\mathbb {C}}[q^{-1}]$, $f\mapsto {\mathcal {P}}_f$, given by the principal part at the cusp at infinity is injective. It follows from Corollary 1 that, for any subfield *K* of ${\mathbb {C}}$, this injection is the ${\mathbb {C}}$-linear extension of the analogous *K*-linear injection $M_{-k}^{!,\infty }(\varGamma _0(N);K) \rightarrow K[q^{-1}]$. Thus, if $f \in M_{-k}^{!,\infty }(\varGamma _0(N))$ is such that ${\mathcal {P}}_f \in K[q^{-1}]\subset {\mathbb {C}}[q^{-1}]$, then *f* itself belongs to $M_{-k}^{!,\infty }(\varGamma _0(N);K) \subset M_{-k}^{!,\infty }(\varGamma _0(N))$. $\square$

## De Rham cohomology with coefficients on modular curves

Let $({\mathcal {V}},\nabla )$ be the *de Rham bundle* over ${\mathcal {Y}}_0(N)$, with its Gauss–Manin connection. Formally, ${\mathcal {V}} :={\mathbb {R}}^1\pi _* \varOmega ^{\bullet }_{{\mathcal {E}}/{\mathcal {Y}}_0(N)}$, where $\pi :{\mathcal {E}} \rightarrow {\mathcal {Y}}_0(N)$ denotes the universal elliptic curve over ${\mathcal {Y}}_0(N)$. If $y: \mathrm {Spec}\, k \rightarrow {\mathcal {Y}}_0(N)$ is a *k*-point corresponding to a pair (*E*, *C*) over *k*, then the fibre of ${\mathcal {V}}$ at *y* is given by the algebraic de Rham cohomology ${\mathcal {V}}(y) = H^1_{\mathrm {dR}}(E/k)$.

One can show (cf. [20] A1.2–A1.3) that ${\mathcal {V}}$ is a rank 2 vector bundle over ${\mathcal {Y}}_0(N)$ containing the Hodge line bundle ${\mathcal {F}}$ as a subbundle, and equipped with a symplectic ${\mathcal {O}}_{{\mathcal {Y}}_0(N)}$-bilinear pairing

$$\begin{aligned} \langle \ , \ \rangle : {\mathcal {V}} \times {\mathcal {V}} \rightarrow {\mathcal {O}}_{{\mathcal {Y}}_0(N)} \end{aligned}$$

induced by the canonical principal polarisation of ${\mathcal {E}}$, satisfying $d\langle v,w\rangle = \langle \nabla v, w \rangle + \langle v, \nabla w \rangle$. For every integer $k\ge 0$, we set

$$\begin{aligned} {\mathcal {V}}_k :={\mathrm {Sym}}^k{\mathcal {V}}\text {,} \end{aligned}$$

and we denote by

$$\begin{aligned} \langle \ , \ \rangle _k: {\mathcal {V}}_k\times {\mathcal {V}}_k \rightarrow {\mathcal {O}}_{{\mathcal {Y}}_0(N)}\text {, }\qquad (\alpha _1\cdots \alpha _k, \beta _1 \cdots \beta _k)\mapsto \frac{1}{k!}\sum _{\sigma \in {\mathfrak {S}}_k}\prod _{j=1}^k\langle \alpha _j,\beta _{\sigma (j)}\rangle \end{aligned}$$

the induced symplectic pairing on ${\mathcal {V}}_k$. By abuse, we continue to denote by $\nabla$ the induced connection on ${\mathcal {V}}_k$.

Consider the ${\mathbb {Q}}$-vector space

$$\begin{aligned} H^1_{\mathrm {dR}}({\mathcal {Y}}_0(N),{\mathcal {V}}_k) :={\mathbb {H}}^1({\mathcal {Y}}_0(N),{\mathcal {V}}_k\otimes \varOmega ^{\bullet }_{{\mathcal {Y}}_0(N)/{\mathbb {Q}}})\text {,} \end{aligned}$$

where ${\mathcal {V}}_k\otimes \varOmega ^{\bullet }_{{\mathcal {Y}}_0(N)/{\mathbb {Q}}}$ is the complex of ${\mathcal {O}}_{{\mathcal {Y}}_0(N)}$-modules concentrated in degrees 0 and 1

$$\begin{aligned}{}[{\mathcal {V}}_k {\mathop {\rightarrow }\limits ^{\nabla }} {\mathcal {V}}_k \otimes \varOmega ^1_{{\mathcal {Y}}_0(N)/{\mathbb {Q}}}]\text {.} \end{aligned}$$

Since ${\mathcal {Y}}_0(N)$ admits an étale cover by a smooth *affine* scheme (Remark 7), we can identify

$$\begin{aligned} H^1_{\mathrm {dR}}({\mathcal {Y}}_0(N),{\mathcal {V}}_k) = \text {coker} (H^0({\mathcal {Y}}_0(N),{\mathcal {V}}_k) {\mathop {\rightarrow }\limits ^{\nabla }}H^0({\mathcal {Y}}_0(N),{\mathcal {V}}_k\otimes \varOmega ^1_{{\mathcal {Y}}_0(N)/{\mathbb {Q}}}))\text {;} \end{aligned}$$

in particular, we have a natural surjection

17 $$\begin{aligned} H^0({\mathcal {Y}}_0(N),{\mathcal {V}}_k\otimes \varOmega ^1_{{\mathcal {Y}}_0(N)/{\mathbb {Q}}}) \twoheadrightarrow H^1_{\mathrm {dR}}({\mathcal {Y}}_0(N),{\mathcal {V}}_k)\text {.} \end{aligned}$$

Recall that there is a *Kodaira–Spencer isomorphism*

$$\begin{aligned} {\mathcal {F}}^{\otimes 2} {\mathop {\rightarrow }\limits ^{\sim }} \varOmega ^1_{{\mathcal {Y}}_0(N)/{\mathbb {Q}}}\text {, } \qquad \omega _1\otimes \omega _2 \mapsto \langle \omega _1,\nabla \omega _2 \rangle \text {,} \end{aligned}$$

whose pullback to ${\mathbb {H}}$ identifies $\omega ^{\otimes 2}$ with $2\pi i\, d\tau = d \log q$ ([20] A1.3.17). Thus,

18 $$\begin{aligned} M_{k+2}^!(\varGamma _0(N);{\mathbb {Q}}) \cong H^0({\mathcal {Y}}_0(N),{\mathcal {F}}^{\otimes k +2}) \cong H^0({\mathcal {Y}}_0(N),{\mathcal {F}}^{\otimes k }\otimes \varOmega ^1_{{\mathcal {Y}}_0(N)/{\mathbb {Q}}})\text {.} \end{aligned}$$

Note that the pullback of $\omega _f$ to the Poincaré half-plane ${\mathbb {H}}$ via (15) gets identified with $f \omega ^{\otimes k} \otimes 2\pi i\, d\tau$. Given the natural inclusion of ${\mathcal {F}}^{\otimes k}$ into ${\mathcal {V}}_k$, we obtain from (17) and (18) a map

$$\begin{aligned} M_{k+2}^!(\varGamma _0(N);{\mathbb {Q}}) \rightarrow H^1_{\mathrm {dR}}({\mathcal {Y}}_0(N),{\mathcal {V}}_k)\text {, }\qquad f\mapsto [\omega _f]=:[f]\text {.} \end{aligned}$$

Recall that we have the holomorphic derivation $D = \frac{1}{2\pi i }\frac{d}{d\tau }$ on ${\mathbb {H}}$. A classical identity of Bol implies that $D^{k+1}f \in M^!_{k+2}(\varGamma _0(N))$ for every $f \in M_{-k}^!(\varGamma _0(N))$ (cf. [8] Proposition 2.2).

### Theorem 4

(cf. [8, 11, 13, 22, 34]) The sequence of ${\mathbb {Q}}$-vector spaces

$$\begin{aligned} 0 \rightarrow M_{-k}^!(\varGamma _0(N);{\mathbb {Q}}) {\mathop {\rightarrow }\limits ^{D^{k+1}}} M^!_{k+2}(\varGamma _0(N);{\mathbb {Q}})&\rightarrow H^1_{\mathrm {dR}}({\mathcal {Y}}_0(N),{\mathcal {V}}_k) \rightarrow 0\\ f&\mapsto [f] \end{aligned}$$

is exact.

Note that $M_{k+2}(\varGamma _0(N);{\mathbb {Q}})$ maps injectively into $H^1_{\mathrm {dR}}({\mathcal {Y}}_0(N),{\mathcal {V}}_k)$.

The above theorem appears to have been rediscovered by several authors. For later reference, we recall the main steps its proof.

### Sketch

We work over *Y*, an affine ${\mathbb {Q}}$-scheme representing ${\mathcal {Y}}_0(N)_p$ for some odd prime *p* not dividing *N* (Remark 7); our statement is then obtained by taking $\mathrm {GL}_2({\mathbb {F}}_p)$-invariants.

Consider the Hodge filtration $F^i{\mathcal {V}}_k :=\mathrm {im}({\mathcal {F}}^{\otimes i}\otimes {\mathcal {V}}_{k-i} \rightarrow {\mathcal {V}}_k)$ on ${\mathcal {V}}_k$. The key fact is that Gauss–Manin connection $\nabla$ induces an isomorphism of ${\mathcal {O}}_Y$-modules

19 $$\begin{aligned} F^1{\mathcal {V}}_k = F^1{\mathcal {V}}_k/F^{k+1}{\mathcal {V}}_k {\mathop {\rightarrow }\limits ^{\sim }} {\mathcal {V}}_k/F^k{\mathcal {V}}_k \otimes \varOmega ^1_{Y/{\mathbb {Q}}}\text {.} \end{aligned}$$

This is a consequence of the Kodaira–Spencer isomorphism, for it implies that $\nabla$ induces the successive isomorphisms $F^i{\mathcal {V}}_k/F^{i+1}{\mathcal {V}}_k {\mathop {\rightarrow }\limits ^{\sim }} (F^{i-1}{\mathcal {V}}_k/ F^{i}{\mathcal {V}}_k)\otimes \varOmega ^1_{Y/{\mathbb {Q}}}$ (cf. [13] Lemma 4.2).

The surjectivity of the natural map

20 $$\begin{aligned} H^0(Y,{\mathcal {F}}^{\otimes k}\otimes \varOmega ^1_{Y/{\mathbb {Q}}}) \rightarrow H^1_{\mathrm {dR}}(Y,{\mathcal {V}}_k) \end{aligned}$$

follows immediately from (19): every $\alpha \in H^0(Y,{\mathcal {V}}_k\otimes \varOmega ^1_{Y/{\mathbb {Q}}})$ can be written as $\alpha = \nabla Q + \beta$ for some $Q \in H^0(Y,F^1{\mathcal {V}}_k)$ and $\beta \in H^0(Y,F^k{\mathcal {V}}_k\otimes \varOmega ^1_{Y/{\mathbb {Q}}}) = H^0(Y,{\mathcal {F}}^k\otimes \varOmega ^1_{Y/{\mathbb {Q}}})$, so that $\alpha$ and $\beta$ map to the same class in $H^1_{\mathrm {dR}}(Y,{\mathcal {V}}_k)$. Its kernel is given by the image of the ${\mathcal {O}}_Y$-morphism

$$\begin{aligned} \delta :{\mathcal {V}}_k/F^1{\mathcal {V}}_k \rightarrow F^k{\mathcal {V}}_k\otimes \varOmega ^1_{Y/{\mathbb {Q}}} = {\mathcal {F}}^{\otimes k}\otimes \varOmega ^1_{Y/{\mathbb {Q}}}\text {, }\qquad [P]\mapsto \nabla P - \nabla Q \end{aligned}$$

where *P* denotes a section of ${\mathcal {V}}_k$, [*P*] its class in ${\mathcal {V}}_k/F^1{\mathcal {V}}_k$, and *Q* the unique section of $F^1{\mathcal {V}}_k$ such that $\nabla P - \nabla Q$ lies in $F^k{\mathcal {V}}_k\otimes \varOmega ^1_{Y/{\mathbb {Q}}}$; the existence and uniqueness of *Q* is guaranteed by the isomorphism (19).

The map $\delta$ is essentially the Bol operator $D^{k+1}$. Indeed, we can identify

$$\begin{aligned} {\mathcal {V}}_k/F^1{\mathcal {V}}_k {\mathop {\rightarrow }\limits ^{\sim }} (F^k{\mathcal {V}}_k)^{\vee }\text {, } \qquad [P]\mapsto \langle \ , P \rangle _k\text {;} \end{aligned}$$

pulling back to ${\mathbb {H}}$, an explicit computation shows that if *f* is a weakly holomorphic modular form of weight $-k$, then $\delta (f\omega ^{\otimes -k}) = \frac{(-1)^k}{k!}D^{k+1}f \omega ^{\otimes k}\otimes 2\pi i \, d\tau$ (cf. [13] Proposition 4.3). $\square$

We now consider cuspidal cohomology. Let $\overline{{\mathcal {V}}} :={\mathbb {R}}^1 {\overline{\pi }}_*\varOmega ^{\bullet }_{\overline{{\mathcal {E}}}/{\mathcal {X}}_0(N)}(\log {\overline{\pi }}^{-1}({\mathcal {Z}}_N))$ with its logarithmic Gauss–Manin connection ${\overline{\nabla }}: \overline{{\mathcal {V}}} \rightarrow \overline{{\mathcal {V}}}\otimes \varOmega ^1_{{\mathcal {X}}_0(N)/{\mathbb {Q}}}(\log {\mathcal {Z}}_N)$. Then, $(\overline{{\mathcal {V}}}, {\overline{\nabla }})$ is a rank 2 vector bundle with logarithmic connection over ${\mathcal {X}}_0(N)$ extending $({\mathcal {V}},\nabla )$. In fact, one can prove that the residues of ${\overline{\nabla }}$ at the cusps are nilpotent (cf. [20] A1.4), so that $(\overline{{\mathcal {V}}},{\overline{\nabla }})$ is Deligne’s canonical extension of $({\mathcal {V}},\nabla )$; in particular,

$$\begin{aligned} H^1_{\mathrm {dR}}({\mathcal {Y}}_0(N),{\mathcal {V}}_k) = {\mathbb {H}}^1({\mathcal {X}}_0(N), \overline{{\mathcal {V}}}_k\otimes \varOmega ^{\bullet }_{{\mathcal {X}}_0(N)/{\mathbb {Q}}}(\log {\mathcal {Z}}_N) )\text {,} \end{aligned}$$

where $\overline{{\mathcal {V}}}_k :=\mathrm {Sym}^k \overline{{\mathcal {V}}}$. The residue $\overline{{\mathcal {V}}}_k\otimes \varOmega ^{1}_{{\mathcal {X}}_0(N)/{\mathbb {Q}}}(\log {\mathcal {Z}}_N) \rightarrow \overline{{\mathcal {V}}}_k|_{{\mathcal {Z}}_N}$ induces a surjective ${\mathbb {Q}}$-linear map

$$\begin{aligned} \mathrm {Res}: H^1_{\mathrm {dR}}({\mathcal {Y}}_0(N),{\mathcal {V}}_k) \rightarrow H^0({\mathcal {Z}}_N,\overline{{\mathcal {V}}}_k|_{{\mathcal {Z}}_N})/\mathrm {im}(\mathrm {Res}{\overline{\nabla }})\cong H^0({\mathcal {Z}}_N,\overline{{\mathcal {F}}}^{\otimes k}|_{{\mathcal {Z}}_N})\text {,} \end{aligned}$$

and we define

$$\begin{aligned} H^1_{\mathrm {dR},\mathrm {cusp}}({\mathcal {Y}}_0(N),{\mathcal {V}}_k) :=\ker ( H^1_{\mathrm {dR}}({\mathcal {Y}}_0(N),{\mathcal {V}}_k) {\mathop {\rightarrow }\limits ^{\mathrm {Res}}} H^0({\mathcal {Z}}_N,\overline{{\mathcal {F}}}^{\otimes k}|_{{\mathcal {Z}}_N}))\text {.} \end{aligned}$$

See [34] 2.6, or [22] 4, for a direct definition of the cuspidal cohomology as the hypercohomology of a subcomplex of $[\overline{{\mathcal {V}}}_k {\mathop {\rightarrow }\limits ^{{\overline{\nabla }}}} \overline{{\mathcal {V}}}_k \otimes \varOmega ^{1}_{{\mathcal {X}}_0(N)/{\mathbb {Q}}}(\log {\mathcal {Z}}_N)]$.

Let $f \in M_{k+2}^!(\varGamma _0(N);{\mathbb {Q}})$. Using the explicit description of the coordinates at the cusps (16), we can identify

$$\begin{aligned} \mathrm {Res}([f]) =(a_{0,g_1}(f),\ldots ,a_{0,g_m}(f))\text {,} \end{aligned}$$

where $\{g_1,\ldots ,g_m\}$ is a set of representatives of $\varGamma _0(N)\backslash \mathrm {SL}_2({\mathbb {Z}})/\varGamma _{\infty }$ (cf. [8], 5B and 5C, for the level 1 case). In particular, the subspace $S_{k+2}^!(\varGamma _0(N);{\mathbb {Q}})\subset M_{k+2}^!(\varGamma _0(N);{\mathbb {Q}})$ maps to the subspace $H^1_{\mathrm {dR},\mathrm {cusp}}({\mathcal {Y}}_0(N),{\mathcal {V}}_k)\subset H^1_{\mathrm {dR}}({\mathcal {Y}}_0(N),{\mathcal {V}}_k)$. It follows from this observation, and from Theorem 4 that the sequence of ${\mathbb {Q}}$-vector spaces

21 $$\begin{aligned} 0 \rightarrow M_{-k}^!(\varGamma _0(N);{\mathbb {Q}}) {\mathop {\rightarrow }\limits ^{D^{k+1}}} S^!_{k+2}(\varGamma _0(N);{\mathbb {Q}})&\rightarrow H^1_{\mathrm {dR},\mathrm {cusp}}({\mathcal {Y}}_0(N),{\mathcal {V}}_k) \rightarrow 0 \end{aligned}$$

is exact.

### Remark 8

The spectral sequence associated to the Hodge filtration induces an exact sequence (cf. proof of Theorem 2.7 in [34])

$$\begin{aligned} 0 \rightarrow S_{k+2}(\varGamma _0(N);{\mathbb {Q}}) \rightarrow H^1_{\mathrm {dR},\mathrm {cusp}}({\mathcal {Y}}_0(N),{\mathcal {V}}_k) \rightarrow S_{k+2}(\varGamma _0(N);{\mathbb {Q}})^{\vee } \rightarrow 0\text {.} \end{aligned}$$

In particular,

$$\begin{aligned} \dim H^1_{\mathrm {dR},\mathrm {cusp}}({\mathcal {Y}}_0(N),{\mathcal {V}}_k) = 2 \dim S_{k+2}(\varGamma _0(N);{\mathbb {Q}})\text {.} \end{aligned}$$

Theorem 4 (and the exact sequence (21)) can be refined: we only need to consider weakly holomorphic cusp forms whose poles are concentrated at the cusp $\infty$. This follows from the next lemma.

### Lemma 5

We have

$$\begin{aligned} M_{k+2}^!(\varGamma _0(N);{\mathbb {Q}}) \subset M_{k+2}^{!,\infty }(\varGamma _0(N);{\mathbb {Q}}) + D^{k+1}M^!_{-k}(\varGamma _0(N);{\mathbb {Q}})\text {.} \end{aligned}$$

### Proof

Let *p* be an odd prime not dividing *N*, and *X* (resp. *Y*) be a ${\mathbb {Q}}$-scheme representing the étale cover ${\mathcal {X}}_0(N)_p$ (resp. ${\mathcal {Y}}_0(N)_p$) of ${\mathcal {X}}_0(N)$ (resp. ${\mathcal {Y}}_0(N)$. Recall that *Y* is affine, and *X* is the projective compactification of *Y* (Remark 7).

We denote by *Z* the cuspidal locus, so that $X\setminus Z = Y$. Consider the affine curve $X^*=X\setminus \{\infty \}$ containing *Y*. We can identify $H^0(X^*, \overline{{\mathcal {F}}}^{\otimes k} \otimes \varOmega ^1_{X/{\mathbb {Q}}}(\log Z))$ as a ${\mathbb {Q}}$-subspace of $H^0(Y, {\mathcal {F}}^{\otimes k} \otimes \varOmega ^1_{Y/{\mathbb {Q}}})$. By taking $\mathrm {GL}_2({\mathbb {F}}_p)$-invariants, our statement will follow from the surjectivity of the natural map

$$\begin{aligned} H^0(X^*, \overline{{\mathcal {F}}}^{\otimes k}\otimes \varOmega ^1_{X/{\mathbb {Q}}}(\log Z)) \rightarrow H^1_{\mathrm {dR}}(Y,{\mathcal {V}}_k)\text {.} \end{aligned}$$

As $(\overline{{\mathcal {V}}}_k,{\overline{\nabla }})|_{X^*}$ is Deligne’s canonical extension of $({\mathcal {V}}_k,\nabla )$ to $X^*$, we have a natural isomorphism

$$\begin{aligned} {\mathbb {H}}^1(X^*,\overline{{\mathcal {V}}}_k\otimes \varOmega ^{\bullet }_{X/{\mathbb {Q}}}(\log Z)){\mathop {\rightarrow }\limits ^{\sim }} H_{\mathrm {dR}}^1(Y,{\mathcal {V}}_k)\text {.} \end{aligned}$$

Since $X^*$ is affine, ${\mathbb {H}}^1(X^*,\overline{{\mathcal {V}}}_k\otimes \varOmega ^{\bullet }_{X/{\mathbb {Q}}}(\log Z))$ gets identified with the cokernel of the *k*-linear map ${\overline{\nabla }}: H^0(X^*,\overline{{\mathcal {V}}}_k) \rightarrow H^0(X^*,\overline{{\mathcal {V}}}_k\otimes \varOmega ^1_{X/{\mathbb {Q}}}(\log Z))$; in particular, we obtain a surjective map

$$\begin{aligned} H^0(X^*,\overline{{\mathcal {V}}}_k\otimes \varOmega ^1_{X/{\mathbb {Q}}}(\log Z)) \rightarrow {\mathbb {H}}^1(X^*,\overline{{\mathcal {V}}}_k\otimes \varOmega ^{\bullet }_{X/{\mathbb {Q}}}(\log Z))\text {.} \end{aligned}$$

The surjectivity of its restriction to the subspace $H^0(X^*, \overline{{\mathcal {F}}}^{\otimes k}\otimes \varOmega ^1_{X/{\mathbb {Q}}}(\log Z))$

$$\begin{aligned} H^0(X^*, \overline{{\mathcal {F}}}^{\otimes k}\otimes \varOmega ^1_{X/{\mathbb {Q}}}(\log Z)) \rightarrow {\mathbb {H}}^1(X^*,\overline{{\mathcal {V}}}_k\otimes \varOmega ^{\bullet }_{X/{\mathbb {Q}}}(\log Z)) \end{aligned}$$

is then proved in the same way as the surjectivity of (20). $\square$

### Corollary 2

The map $M^!_{k+2}(\varGamma _0(N);{\mathbb {Q}}) \rightarrow H^1_{\mathrm {dR}}({\mathcal {Y}}_0(N),{\mathcal {V}}_k)$, $f\mapsto [f]$ induces short exact sequences of ${\mathbb {Q}}$-vector spaces

$$\begin{aligned} 0 \rightarrow M_{-k}^{!,\infty }(\varGamma _0(N);{\mathbb {Q}}) {\mathop {\rightarrow }\limits ^{D^{k+1}}} M^{!,\infty }_{k+2}(\varGamma _0(N);{\mathbb {Q}}) \rightarrow H^1_{\mathrm {dR}}({\mathcal {Y}}_0(N),{\mathcal {V}}_k) \rightarrow 0 \end{aligned}$$

and

$$\begin{aligned} 0 \rightarrow M_{-k}^{!,\infty }(\varGamma _0(N);{\mathbb {Q}}) {\mathop {\rightarrow }\limits ^{D^{k+1}}} S^{!,\infty }_{k+2}(\varGamma _0(N);{\mathbb {Q}}) \rightarrow H^1_{\mathrm {dR},\mathrm {cusp}}({\mathcal {Y}}_0(N),{\mathcal {V}}_k) \rightarrow 0\text {.} \end{aligned}$$

$$\square $$

We finish this section with a brief discussion of the de Rham pairing

22 $$\begin{aligned} \langle \ , \ \rangle _{\mathrm {dR}} : H^1_{\mathrm {dR},\mathrm {cusp}}({\mathcal {Y}}_0(N),{\mathcal {V}}_k)\times H^1_{\mathrm {dR}}({\mathcal {Y}}_0(N),{\mathcal {V}}_k) \rightarrow {\mathbb {Q}}\text {,} \end{aligned}$$

whose restriction to $H^1_{\mathrm {dR},\mathrm {cusp}}({\mathcal {Y}}_0(N),{\mathcal {V}}_k)\times H^1_{\mathrm {dR},\mathrm {cusp}}({\mathcal {Y}}_0(N),{\mathcal {V}}_k)$ is a symplectic ${\mathbb {Q}}$-bilinear form.

Explicitly, we can compute $\langle \ , \ \rangle _{\mathrm {dR}}$ (defined in terms of the cup product in de Rham cohomology) via the usual recipe involving residues at the cusps (cf. [13] Theorem 5.2). In fact, by Lemma 5, we only need to consider the cusp $\infty$. Let $\varphi : \mathrm {Spec}\, {\mathbb {Q}}(\!(q)\!) \rightarrow {\mathcal {Y}}_0(N)$ be the morphism given by the Tate curve (as in the proof of Proposition 5). For $f \in S_{k+2}^{!,\infty }(\varGamma _0(N);{\mathbb {Q}})$ and $g \in M_{k+2}^{!,\infty }(\varGamma _0(N);{\mathbb {Q}})$, we have

$$\begin{aligned} \langle [f],[g]\rangle _{\mathrm {dR}} = {\mathrm {Res}}_{q=0}\langle F,\varphi ^*\omega _g \rangle _k\text {,} \end{aligned}$$

where $$F \in H^0(\mathrm {Spec}\, {\mathbb {Q}}(\!(q)\!), \varphi ^*{\mathcal {V}}_k)$$ satisfies

$$\begin{aligned} \nabla F = \varphi ^*\omega _f = f(q)\omega ^{k}\otimes d \log q\text {.} \end{aligned}$$

Solving for *F*, we obtain the explicit formula

23 $$\begin{aligned} \langle [f],[g]\rangle _{\mathrm {dR}} = k!\sum _{n \in {\mathbb {Z}}}\frac{a_n(f)a_{-n}(g)}{n^{k+1}}\text {.} \end{aligned}$$

Note that, since *g* is meromorphic at infinity, the above sum is finite.

## Realisations of modular motives

Next, we construct an object $H^1({\mathcal {Y}}_0(N),V_k)$ of ${\mathcal {H}}({\mathbb {Q}})$ whose de Rham realisation is

$$\begin{aligned} H^1({\mathcal {Y}}_0(N),V_k)_{\mathrm {dR}} = H^1_{\mathrm {dR}}({\mathcal {Y}}_0(N),{\mathcal {V}}_k)\text {,} \end{aligned}$$

as defined in the last section. Throughout, $k\ge 0$ is an *even* integer.

For the Betti realisation, let $\pi : {\mathcal {E}} \rightarrow {\mathcal {Y}}_0(N)$ be the universal elliptic curve over ${\mathcal {Y}}_0(N)$, and consider the ${\mathbb {Q}}$-local system

$$\begin{aligned} {\mathbb {V}} :=R^1\pi ^{\mathrm {an}}_{{\mathbb {C}},*} {\mathbb {Q}}_{{\mathcal {E}}^{\mathrm {an}}_{{\mathbb {C}}}} \end{aligned}$$

over ${\mathcal {Y}}_0(N)_{{\mathbb {C}}}^{\mathrm {an}}$, whose stalk at a point *y* of ${\mathcal {Y}}_0(N)_{{\mathbb {C}}}^{\mathrm {an}}$, corresponding to a pair (*E*, *C*) defined over ${\mathbb {C}}$, is the Betti cohomology with rational coefficients ${\mathbb {V}}_y = H^1(E^{\mathrm {an}},{\mathbb {Q}})$. Setting ${\mathbb {V}}_k :=\mathrm {Sym}^k {\mathbb {V}}$, we define

$$\begin{aligned} H^1({\mathcal {Y}}_0(N),V_k)_{\mathrm {B}} = H^1({\mathcal {Y}}_0(N)_{{\mathbb {C}}}^{\mathrm {an}},{\mathbb {V}}_k)\text {.} \end{aligned}$$

The Betti realisation has a concrete description in terms of group cohomology. The pullback of ${\mathbb {V}}$ to ${\mathbb {H}}$ via the uniformisation map ${\mathbb {H}} \rightarrow {\mathcal {Y}}_0(N)_{{\mathbb {C}}}^{\mathrm {an}}$ (15) is trivialised by the global sections

$$\begin{aligned} X:=\sigma _1^{\vee }\ \ \ \text { and }\ \ \ Y :=-\sigma _{\tau }^{\vee }\text {,} \end{aligned}$$

where $\sigma _1,\sigma _{\tau } \in H^0({\mathbb {H}},{\mathbb {V}}^{\vee })$ are the constant families of 1-cycles given by 1 and $\tau$ under the identification $H^1({\mathbb {E}}_{\tau },{\mathbb {Q}})^{\vee }={\mathbb {Q}} + \tau {\mathbb {Q}}$. Thus, ${\mathbb {V}}$ can be regarded as the vector space $V = {\mathbb {Q}}X \oplus {\mathbb {Q}} Y$ endowed with the right $\varGamma _0(N)$-action

$$\begin{aligned} (X,Y)|_{\gamma } = (aX + bY,cX + dY)\text {, } \qquad \gamma = \left( \begin{array}{cc} a &{} b \\ c &{} d\end{array} \right) \in \varGamma _0(N)\text {.} \end{aligned}$$

In particular, if we set $V_k :=\mathrm {Sym}^k V_k$, then we have a canonical identification

24 $$\begin{aligned} H^1({\mathcal {Y}}_0(N)_{{\mathbb {C}}}^{\mathrm {an}},{\mathbb {V}}_k) \cong H^1(\varGamma _0(N),V_k)\text {.} \end{aligned}$$

Fixing an arbitrary $\tau _0\in {\mathbb {H}}$, the comparison isomorphism

25 $$\begin{aligned} \mathrm {comp}: H^1_{\mathrm {dR}}({\mathcal {Y}}_0(N),{\mathcal {V}}_k) \otimes _{{\mathbb {Q}}} {\mathbb {C}} {\mathop {\rightarrow }\limits ^{\sim }} H^1({\mathcal {Y}}_0(N)_{{\mathbb {C}}}^{\mathrm {an}},{\mathbb {V}}_k)\otimes _{{\mathbb {Q}}}{\mathbb {C}} \end{aligned}$$

can be explicitly given by

$$\begin{aligned} \mathrm {comp}([f]) = [C_f]\text {,} \end{aligned}$$

where [*f*] denotes the class of some $f \in M_{k+2}^!(\varGamma _0(N))$ (see Sect. 5), and $C_f : \varGamma _0(N) \rightarrow V_k$ is the 1-cocycle defined by

26 $$\begin{aligned} C_{f}:\varGamma _0(N) \rightarrow V_k\text {, }\qquad \gamma \mapsto (2\pi i)^{k+1}\int _{\gamma ^{-1}\cdot \tau _0}^{\tau _0}f(\tau )(X - \tau Y)^kd\tau \text {.} \end{aligned}$$

This follows from the fact that the ‘relative comparison isomorphism’

27 $$\begin{aligned} \mathrm {comp}: ({\mathcal {V}}_{{\mathbb {C}}}^{\mathrm {an}},\nabla ^{\mathrm {an}}) {\mathop {\rightarrow }\limits ^{\sim }} ({\mathbb {V}}\otimes _{{\mathbb {Q}}} {\mathcal {O}}_{{\mathcal {Y}}_0(N)^{\mathrm {an}}_{{\mathbb {C}}}},\mathrm {id}\otimes d) \end{aligned}$$

satisfies

$$\begin{aligned} \mathrm {comp}(\omega ) = \left( \int _{\sigma _1}\omega \right) \sigma _1^{\vee } + \left( \int _{\sigma _{\tau }}\omega \right) \sigma _\tau ^{\vee } = 2\pi i (X - \tau Y)\text {.} \end{aligned}$$

The weight and Hodge filtrations on $H^1_{\mathrm {dR}}({\mathcal {Y}}_0(N),{\mathcal {V}}_k)$ are explicitly defined by

$$\begin{aligned}&W^{\mathrm {dR}}_{k} = 0,\\&W^{\mathrm {dR}}_{k+1} = \cdots = W^{\mathrm {dR}}_{2k+1} = H^1_{\mathrm {dR},\mathrm {cusp}}({\mathcal {Y}}_0(N),{\mathcal {V}}_k),\\&W^{\mathrm {dR}}_{2k+2} = H^1_{\mathrm {dR}}({\mathcal {Y}}_0(N),{\mathcal {V}}_k) \end{aligned}$$

and

$$\begin{aligned}&F_{\mathrm {dR}}^0 = H_{\mathrm {dR}}^1({\mathcal {Y}}_0(N),{\mathcal {V}}_k),\\&F^1_{\mathrm {dR}} = \cdots = F_{\mathrm {dR}}^{k+1} = \mathrm {im}(H^0({\mathcal {X}}_0(N),\overline{{\mathcal {F}}}^{\otimes k}\otimes \varOmega _{{\mathcal {X}}_0(N)/{\mathbb {Q}}}^1(\log {\mathcal {Z}}_N))\rightarrow H_{\mathrm {dR}}^1({\mathcal {Y}}_0(N),{\mathcal {V}}_k)),\\&F_{\mathrm {dR}}^{k+2}=0\text {.} \end{aligned}$$

The weight filtration on $H^1({\mathcal {Y}}_0(N)_{{\mathbb {C}}}^{\mathrm {an}},{\mathbb {V}}_k)$ is defined by

$$\begin{aligned}&W^{\mathrm {B}}_k=0,\\&W^{\mathrm {B}}_{k+1} = \cdots = W^{\mathrm {B}}_{2k+1} = \mathrm {im}(H^1({\mathcal {X}}_0(N)_{{\mathbb {C}}}^{\mathrm {an}}, j^{\mathrm {an}}_{{\mathbb {C}},*}{\mathbb {V}}_k)\rightarrow H^1({\mathcal {Y}}_0(N)_{{\mathbb {C}}}^{\mathrm {an}},{\mathbb {V}}_k)),\\&W^{\mathrm {B}}_{2k+2} = H^1({\mathcal {Y}}_0(N)_{{\mathbb {C}}}^{\mathrm {an}},{\mathbb {V}}_k)\text {,} \end{aligned}$$

where $j: {\mathcal {Y}}_0(N) \rightarrow {\mathcal {X}}_0(N)$ is the natural open immersion. The real Frobenius $F_{\infty }$ is induced by the complex conjugation ${\mathcal {Y}}_0(N)_{{\mathbb {C}}}^{\mathrm {an}} \rightarrow {\mathcal {Y}}_0(N)_{{\mathbb {C}}}^{\mathrm {an}}$; under the identification (24), we have

$$\begin{aligned} F_{\infty }([C_f]) = [\gamma \mapsto C_f(\epsilon \gamma \epsilon ^{-1})|_{\epsilon }]\text {, }\qquad \epsilon = \left( \begin{array}{cc} 1 &{} 0 \\ 0 &{}-1\end{array}\right) \text {.} \end{aligned}$$

### Remark 9

Under the identification (24), the subspace $W^{\mathrm {B}}_{k+1}$ is given by the parabolic cohomology $\bigcap _{c}\ker (H^1(\varGamma _0(N),V_k) \rightarrow H^1(\varGamma _0(N)_c,V_k))$, where *c* runs through the set of cusps of $\varGamma _0(N)$ and $\varGamma _0(N)_c\le \varGamma _0(N)$ denotes the stabiliser of the cusp *c* (see [37] Proposition 12.5).

### Theorem 5

With the above definitions, the triple

$$\begin{aligned} H^1({\mathcal {Y}}_0(N),V_k) :=((H^1({\mathcal {Y}}_0(N),V_k)_{\mathrm {B}}, W^{\mathrm {B}}, F_{\infty }),(H^1({\mathcal {Y}}_0(N),V_k)_{\mathrm {dR}},W^{\mathrm {dR}},F_{\mathrm {dR}}),\mathrm {comp})\text {,} \end{aligned}$$

is an object of the category of realisations ${\mathcal {H}}({\mathbb {Q}})$. Moreover, the weight $k+1$ part of $H^1({\mathcal {Y}}_0(N),V_k)$, which we denote by $H_{\mathrm {cusp}}^1({\mathcal {Y}}_0(N),V_k)$, is of Hodge type $\{(k+1,0),(0,k+1)\}$.

Note that the Hodge filtration $F^{k+1}_{\mathrm {dR}}$ on $H^1_{\mathrm {dR}}({\mathcal {Y}}_0(N),{\mathcal {V}}_k)$ (resp. on $H^1_{\mathrm {dR},\mathrm {cusp}}({\mathcal {Y}}_0(N),{\mathcal {V}}_k)$) is isomorphic to $M_{k+2}(\varGamma _0(N);{\mathbb {Q}})$ (resp. $S_{k+2}(\varGamma _0(N);{\mathbb {Q}})$) via the map $f\mapsto [f]$ of Theorem 4.

### Proof

The commutativity of the diagram (7) can be readily verified from the above explicit definitions of $\mathrm {comp}$ and $F_{\infty }$. That $(H^1({\mathcal {Y}}_0(N),V_k)_{\mathrm {B}}, W^{\mathrm {B}}, \mathrm {comp}(F_{\mathrm {dR}}))$ is a ${\mathbb {Q}}$-mixed Hodge structure follows from a theorem of Zucker [37] (Sections 12 and 13). Finally, that $H_{\mathrm {cusp}}^1({\mathcal {Y}}_0(N),V_k)$ is of Hodge type $\{(k+1,0),(0,k+1)\}$ is the content of the classical Eichler–Shimura isomorphism (see also [37] Section 12). $\square$

The pure object $H^1_{\mathrm {dR},\mathrm {cusp}}({\mathcal {Y}}_0(N),{\mathcal {V}}_k)$ of ${\mathcal {H}}({\mathbb {Q}})$ admits a canonical polarisation

$$\begin{aligned} \langle \ , \ \rangle : H^1_{\mathrm {cusp}}({\mathcal {Y}}_0(N),V_k) \times H^1_{\mathrm {cusp}}({\mathcal {Y}}_0(N),V_k) \rightarrow {\mathbb {Q}}(-k-1) \end{aligned}$$

whose de Rham realisation $\langle \ , \ \rangle _{\mathrm {dR}}$ was defined at the end of Sect. 5. The Betti realisation $\langle \ , \ \rangle _{\mathrm {B}}$ is defined in terms of the cup product in the same way; here, we use the ${\mathbb {Q}}$-linear pairing of local systems $\langle \ , \ \rangle :{\mathbb {V}} \times {\mathbb {V}} \rightarrow {\mathbb {Q}}_{{\mathcal {Y}}_0(N)_{{\mathbb {C}}}^{\mathrm {an}}}$ whose fibres are induced by the usual intersection pairing on $H_1(E^{\mathrm {an}},{\mathbb {Z}})$, where *E* is a complex elliptic curve. Explicitly, over ${\mathbb {H}}$, we have

28 $$\begin{aligned} \langle X , Y \rangle = -1\text {.} \end{aligned}$$

Let us now consider the de Rham Hermitian form $( \ , \ )_{\mathrm {dR}}$ on the vector space $H^1_{\mathrm {dR},\mathrm {cusp}}({\mathcal {Y}}_0(N),{\mathcal {V}}_k)\otimes _{{\mathbb {Q}}}{\mathbb {C}}$ as defined in Sect. 2. The next proposition shows that its restriction to $S_{k+2}(\varGamma _0(N))$ is, up to an explicit factor, the usual Petersson inner product of cusp forms.

### Proposition 6

Consider the injection $f\mapsto [f]$ of $S_{k+2}(\varGamma _0(N))$ into $H^1_{\mathrm {dR},\mathrm {cusp}}({\mathcal {Y}}_0(N),{\mathcal {V}}_k)\otimes _{{\mathbb {Q}}}{\mathbb {C}}$ of Sect. 5. For every $f,g \in S_{k+2}(\varGamma _0(N))$, we have

$$\begin{aligned} ( [f] , [g] )_{\mathrm {dR}} = (4\pi )^{k+1}\int _{\varGamma _0(N)\backslash {\mathbb {H}}}f(\tau )\overline{g(\tau )} y^{k+2}\frac{dx\wedge dy}{y^2} \end{aligned}$$

where $$\tau = x + i y$$.

### Proof

We work in $C^{\infty }$ de Rham cohomology. Note that the real structure coming from Betti cohomology coincides with the real structure coming from real valued $C^{\infty }$ de Rham cohomology, so that, by Lemma 1, $\mathrm {sv}\otimes c_{\mathrm {dR}}$ acts as the usual complex conjugation of complex valued $C^{\infty }$ differential forms. Since [*f*] is represented by $f\omega ^k\otimes (2\pi i d\tau )$, and similarly for [*g*], it follows from the construction of the pairing $\langle \ , \ \rangle _{\mathrm {dR}}$ in terms of the de Rham cup product that

$$\begin{aligned} ( [f] , [g] )_{\mathrm {dR}} = (-1)^{k+1}\frac{1}{2\pi i}\int _{\varGamma _0(N)\backslash {\mathbb {H}}}f(\tau )\overline{g(\tau )}\langle \omega ^k, {\overline{\omega }}^k\rangle _k (2\pi i d\tau ) \wedge (-2\pi i d {\overline{\tau }}). \end{aligned}$$

We compute the de Rham pairing $\langle \omega , {\overline{\omega }}\rangle$ in terms of the Betti pairing on ${\mathbb {V}}$ by using the relative comparison isomorphism (27) and Eq. (28):

$$\begin{aligned} \langle \omega ,{\overline{\omega }}\rangle&= \frac{1}{2\pi i}\langle \mathrm {comp}(\omega ), \overline{\mathrm {comp}(\omega }) \rangle = \frac{1}{2\pi i}\langle 2\pi i (X-\tau Y),-2\pi i (X-{{\overline{\tau }}} Y) \rangle \\&= -2\pi i ({\overline{\tau }} - \tau ) = -4\pi y. \end{aligned}$$

Thus, $\langle \omega ^k,{\overline{\omega }}^k\rangle _k = (-4\pi y)^k$ and the statement follows. $\square$

### Remark 10

Although we do not work explicitly with Betti realisations in this paper, let us remark that, under the identification (24), one can relate the Betti pairing $\langle \ , \ \rangle _{\mathrm {B}}$ with a generalisation of Harberland’s inner product. Then, the Haberland formula (see [26] Theorem 3.2), relating this inner product to the Petersson inner product, follows immediately from the commutativity of (10) and from the above proposition.

A classical computation relying on the orthonormality of $\{e^{2\pi i n x}\}_{n\in {\mathbb {Z}}}$ in $L^2([0,1])$ yields (cf. [12] Theorem 8.2.3, [30] Theorem 5.7.3):

### Corollary 3

Let $m\ge 1$ be an integer, and $f \in S_{k+2}(\varGamma _0(N))$ be a cusp form. Then,

$$\begin{aligned} ([f],[P_{m,k+2,N}])_{\mathrm {dR}} = \frac{k!}{m^{k+1}}a_m(f)\text {.} \end{aligned}$$

Let us recall that the above formula implies in particular that the Poincaré series $P_{m,k+2,N}$ with $m\ge 1$ generate the (finite-dimensional) vector space $S_{k+2}(\varGamma _0(N))$; this will be used throughout in Sect. 9.

## Harmonic Maass forms and the single-valued involution

Next, we relate the single-valued involution on the objects $H^1({\mathcal {Y}}_0(N),V_k)$ of ${\mathcal {H}}({\mathbb {Q}})$ constructed in Sect. 6 with the theory of harmonic Maass forms of integral weight, which we now briefly recall.

The hyperbolic Laplacian of weight $r\in {\mathbb {Z}}$ is the differential operator on ${\mathbb {H}}$ defined by

$$\begin{aligned} \varDelta _r = - y\left( \frac{\partial ^2}{\partial x^2} + \frac{\partial ^2}{\partial y^2} \right) + iry\left( \frac{\partial }{\partial x} + i\frac{\partial }{\partial y} \right) \text {, } \end{aligned}$$

where $$\tau = x+iy$$.

### Definition 6

**(cf. **[4] **4.1)** A *harmonic Maass form of manageable growth* of weight *r* and level $\varGamma _0(N)$ is a $C^{\infty }$ function $F: {\mathbb {H}} \rightarrow {\mathbb {C}}$ which is modular of weight *r* for $\varGamma _0(N)$:

$$\begin{aligned} F|_{\gamma ,r}=F \end{aligned}$$

for every $\gamma \in \varGamma _0(N)$, harmonic:

$$\begin{aligned} \varDelta _rF = 0\text {,} \end{aligned}$$

and has ‘manageable growth’ at all cusps: for every $g \in \mathrm {SL}_2({\mathbb {Z}})$, there exists $\rho >0$ such that

$$\begin{aligned} F|_{g,r} = O(e^{\rho \mathfrak {I}\tau }) \end{aligned}$$

as $$\mathfrak {I}\tau \rightarrow +\infty $$.

The space of harmonic Maass forms of manageable growth of weight *r* and level $\varGamma _0(N)$ is denoted by $H_r^!(\varGamma _0(N))$. If the last condition above is replaced by “for every $g \in \mathrm {SL}_2({\mathbb {Z}})$, there exists $P \in {\mathbb {C}}[q^{-1}]$ and $\rho >0$ such that

$$\begin{aligned} F|_{g,r}-P(e^{2\pi i \tau }) = O(e^{-\rho \mathfrak {I}\tau }) \end{aligned}$$

as $\mathfrak {I}\tau \rightarrow +\infty$”, then we say that *F* is a *harmonic Maass form* of weight *r* and level $\varGamma _0(N)$. The subspace of harmonic Maass forms is denoted by $H_r(\varGamma _0(N))\subset H_r^!(\varGamma _0(N))$; it contains the space $M^!_{r}(\varGamma _0(N))$ of weakly holomorphic modular forms of weight *r* and level $\varGamma _0(N)$.

The following result summarises the main properties of harmonic Maass forms of manageable growth with respect to the differential operators $\xi _{-k}$ and $D^{k+1}$, defined on a smooth function $F:{\mathbb {H}} \rightarrow {\mathbb {C}}$ by

$$\begin{aligned} \xi _{-k}(F) = 2i (\mathfrak {I}\tau )^{-k} \overline{\frac{\partial F}{\partial {\overline{\tau }}}} \text {, }\ \ \ D^{k+1}(F) = \frac{1}{(2\pi i)^{k+1}}\frac{\partial ^{k+1}F}{\partial \tau ^{k+1}}\text {.} \end{aligned}$$

### Theorem 6

([4, 9, 10] Chapter 5) Let $k \ge 0$ be an even integer, and $F \in H_{-k}^!(\varGamma _0(N))$. Then, $\xi _{-k}F$ and $D^{k+1}F$ belong to $M_{k+2}^!(\varGamma _0(N))$. Moreover, $F \in H_{-k}(\varGamma _0(N))$ if and only if $\xi _{-k}F \in S_{k+2}(\varGamma _0(N))$. Finally, the following sequence of ${\mathbb {C}}$-vector spaces 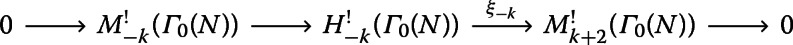 is exact.

To state our next theorem, we introduce the following notion, which will also be used in the following sections.

### Definition 7

We say that $f,g \in M_{k+2}^!(\varGamma _0(N))$ are *Betti conjugate* if

$$\begin{aligned} (\mathrm {sv}\otimes c_{\mathrm {dR}})([f]) = [g] \end{aligned}$$

in $$H^1_{\mathrm {dR}}({\mathcal {Y}}_0(N),{\mathcal {V}}_k)\otimes _{{\mathbb {Q}}}{\mathbb {C}}$$.

Our terminology is justified by Lemma 1, which implies that *f* and *g* are Betti conjugate if and only if their classes in Betti cohomology are complex conjugate:

$$\begin{aligned} (\mathrm {id}\otimes c_{\mathrm {B}})(\mathrm {comp}([f])) = \mathrm {comp}([g])\text {.} \end{aligned}$$

### Theorem 7

Let $k\ge 0$ be an even integer, and $f,g \in M_{k+2}^!(\varGamma _0(N))$ be Betti conjugate. Then, there exists $F \in H_{-k}^!(\varGamma _0(N))$ such that

$$\begin{aligned} \xi _{-k}F = (4\pi )^{k+1}f\ \ \text { and }\ \ D^{k+1}F = k! g\text {.} \end{aligned}$$

If $k=0$, then *F* is unique up to an additive constant; otherwise, *F* is unique.

This theorem is essentially equivalent to the results of Brown [6] when $N=1$, and of Candelori [11] when $N\ge 5$ and *f* is a cusp form. We prove it below for completeness; our approach is similar to Candelori’s.

### Proof

Recall that working over ${\mathcal {Y}}_0(N)_{{\mathbb {C}}}^{\mathrm {an}}$ amounts to working over ${\mathbb {H}}$ equivariantly under the left action of $\varGamma _0(N)$. This will be implicit in what follows.

After the Kodaira–Spencer identification ${\mathcal {F}}^{\otimes 2} \cong \varOmega ^1_{{\mathcal {Y}}_0(N)/{\mathbb {Q}}}$, we have

$$\begin{aligned} \omega _f = f\omega ^k \otimes 2\pi i\, d\tau \in H^0({\mathcal {Y}}_0(N)_{{\mathbb {C}}}^{\mathrm {an}}, {\mathcal {V}}^{\mathrm {an}}_k\otimes \varOmega ^1_{{\mathcal {Y}}_0(N)_{{\mathbb {C}}}^{\mathrm {an}}})\text {,} \end{aligned}$$

and similarly for $\omega _g$. As the real structure on the complex analytic de Rham cohomology $H^1_{\mathrm {dR}}({\mathcal {Y}}_0(N)_{{\mathbb {C}}}^{\mathrm {an}},{\mathcal {V}}_{k}^{\mathrm {an}})$ coming from real valued $C^{\infty }$ de Rham cohomology coincides with the real structure given by the Betti cohomology $H^1({\mathcal {Y}}_0(N)_{{\mathbb {C}}}^{\mathrm {an}},{\mathbb {V}}_k)\otimes _{{\mathbb {Q}}} {\mathbb {R}}$ after the natural comparison isomorphisms (cf. proof of Proposition 6), the image of the $C^{\infty }$ differential form with coefficients in ${\mathcal {V}}^{\mathrm {an}}_k$

$$\begin{aligned} \omega _{g}-\overline{\omega _f} = g\omega ^k\otimes 2\pi i\, d\tau + {\overline{f}}{\overline{\omega }}^k\otimes 2\pi i\, d{\overline{\tau }} \end{aligned}$$

in $H^1_{\mathrm {dR}}({\mathcal {Y}}_0(N)_{{\mathbb {C}}}^{\mathrm {an}}, {\mathcal {V}}_k^{\mathrm {an}})$ is zero by Lemma 1. Thus, there exists a $C^{\infty }$ global section *A* of ${\mathcal {V}}_k^{\mathrm {an}}$ satisfying

$$\begin{aligned} \nabla A = \omega _{g}-\overline{\omega _f} \text {,} \end{aligned}$$

where $\nabla$ denotes the Gauss–Manin connection on ${\mathcal {V}}_k^{\mathrm {an}}$. Since $\omega$ and ${\overline{\omega }}$ trivialise ${\mathcal {V}}^{\mathrm {an}}$ over ${\mathbb {H}}$, we can uniquely write

$$\begin{aligned} A = \sum _{r+s=k}A_{r,s} \omega ^r {\overline{\omega }}^s\text {,} \end{aligned}$$

where $A_{r,s}$ are $C^{\infty }$ functions on ${\mathbb {H}}$. We set

$$\begin{aligned} F :=(-4\pi i \mathfrak {I}\tau )^k A_{0,k}\text {.} \end{aligned}$$

To check that *F* satisfies $\xi _{-k}F = (4\pi )^{k+1}f$ and $D^{k+1}F=k!g$, it is convenient to use Brown’s terminology and notation in [6]. Note first that, by $\varGamma _0(N)$-invariance of *A*, the $C^{\infty }$ functions $A_{r,s}$ are modular of weights (*r*, *s*) for $\varGamma _0(N)$. In particular, since

$$\begin{aligned} 2{\mathbb {L}} :=-4\pi \mathfrak {I}\tau \end{aligned}$$

is modular of weights $(-1,-1)$, the function *F* is modular of weights $(-k,0)$.

By definition of *A*, we have

29 $$\begin{aligned} \nabla _DA = g\omega ^k\text {.} \end{aligned}$$

Using the formulas

$$\begin{aligned} \nabla _D\omega = \frac{\omega + {\overline{\omega }}}{2{\mathbb {L}}}\text {, } \ \ \ \nabla _{D}{\overline{\omega }} = 0\text {,} \end{aligned}$$

which can be checked by computing the periods along the 1-cycles $\sigma _1$ and $\sigma _\tau$, we obtain from (29) the equations

$$\begin{aligned} \partial _k A_{k,0} = 2{\mathbb {L}}g\ \ \ \text { and }\ \ \ \partial _rA_{r,k-r} + (r+1)A_{r+1,k-r-1} = 0\text {, }\qquad 0\le r < k\text {,} \end{aligned}$$

where $\partial _r :=2{\mathbb {L}}D + r$. Thus, ([6] Lemmas 3.2 and 3.3)

$$\begin{aligned} D^{k+1}F = \frac{1}{(2{\mathbb {L}})^{k+1}}\partial _0 \partial _{-1} \cdots \partial _{-k}F = \partial _k \partial _{k-1}\cdots \partial _0 A_{0,k} = (-1)^kk!g = k!g\text {.} \end{aligned}$$

Using that

$$\begin{aligned} \nabla _{{\overline{D}}}A = -{\overline{f}} {\overline{\omega }}^k \end{aligned}$$

we conclude similarly that $\xi _{-k}F = (4\pi )^{k+1}f$.

Finally, we check that *F* is a harmonic Maass form of manageable growth of weight $-k$ and level $\varGamma _0(N)$. The modularity property has already been remarked above. That *F* is harmonic follows from the formula $\varDelta _{-k} = -\partial _{-k-1}{\overline{\partial }}_0$ and from the fact that $\xi _{-k}F = (4\pi )^{k+1}f$ is holomorphic. The growth condition at the cusps follows from those of *f* and *g*, and from the equations $D^{k+1}F = k! g$ and $\xi _{-k}F = (4\pi )^{k+1}f$; this can be seen either via the Fourier expansion of *F* as in [9] (Section 3), or via an explicit expression of *F* in terms of *f* and *g* as in [6] (Section 5.3). $\square$

### Corollary 4

The following diagram of ${\mathbb {C}}$-vector spaces commutes: 
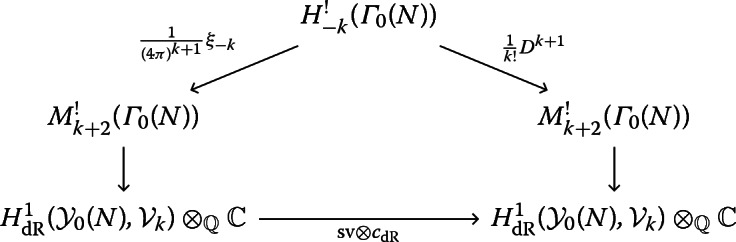
 where the vertical arrows are the quotient maps $f\mapsto [f]$ of Theorem 4. $\square$

### Remark 11

In particular, we obtain a formula for the de Rham pairing $( \ , \ )_{\mathrm {dR}}$ defined in Lemma 3 in terms of harmonic lifts. Namely, let $f,g \in M_{k+2}^{!,\infty }(\varGamma _0(N))$, and consider any $G \in H^{!}_{-k}(\varGamma _0(N))$ such that $\xi _{-k}G = (4\pi )^{k+1}g$. Then, by (23)

$$\begin{aligned} ([f],[g])_{\mathrm {dR}}&= -\langle [f], (\mathrm {sv}\otimes c_{\mathrm {dR}})([g])\rangle _{\mathrm {dR}}= -\frac{1}{k!}\langle [f], [D^{k+1}G]\rangle _{\mathrm {dR}}\\&= \sum _{n\in {\mathbb {Z}}}\frac{a_n(f)a_{-n}(D^{k+1}G)}{n^{k+1}}= \sum _{n\in {\mathbb {Z}}}a_n(f)a_{-n}(G^+) \end{aligned}$$

where $G^+$ is the holomorphic part of *G* (see [4] Section 4.2). This provides a natural cohomological interpretation for the ‘regularised Petersson inner product’ (cf. [3] Theorem 1.1).

As an application of Corollary 4, we have the following result on Poincaré series.

### Proposition 7

Let $k\ge 0$ be an even integer. For every integer $m>0$, the Poincaré series $P_{m,k+2,N}$ and $-P_{-m,k+2,N}$ are Betti conjugate.

Actually, since Poincaré series have real Fourier coefficients at infinity (Proposition 3), we have

$$\begin{aligned} \mathrm {sv}([P_{m,k+2,N}]) = - [P_{-m,k+2,N}] \end{aligned}$$

in $H^1_{\mathrm {dR}}({\mathcal {Y}}_0(N), {\mathcal {V}}_k)\otimes _{{\mathbb {Q}}}{\mathbb {R}}$. We remark that, in level 1, this is consistent with the action of $\mathrm {sv}$ on Eisenstein series in [6], which corresponds to the case $m=0$ in our notation.

### Proof

We use the following result of Bringmann and Ono [2] (see also [10]): there exists a harmonic Maass form $Q_{-m,k+2,N} \in H^!_{-k}(\varGamma _0(N))$, the so-called *Maass–Poincaré series*, satisfying

$$\begin{aligned} \xi _{-k}Q_{-m,k+2,N}= \frac{(4\pi )^{k+1}m^{k+1}}{k!}P_{m,k+2,N} \end{aligned}$$

and

$$\begin{aligned} D^{k+1}Q_{-m,k+2,N} = -m^{k+1}P_{-m,k+2,N}\text {.} \end{aligned}$$

By considering $\frac{k!}{m^{k+1}}Q_{-m,k+2,N}\in H^!_{-k}(\varGamma _0(N))$, our statement follows immediately from Corollary 4. $\square$

## Coefficients of Poincaré series as single-valued periods: rank 2 case

Let $k\ge 0$ and $N\ge 1$ be integers such that $\dim S_{k+2}(\varGamma _0(N))=1$, so that

$$\begin{aligned} \dim H^1_{\mathrm {dR},\mathrm {cusp}}({\mathcal {Y}}_0(N),{\mathcal {V}}_k) = 2\text {.} \end{aligned}$$

This only happens in the finite number of cases given by Table 1 below (see [36]):

**Table 1:** Cases where $\dim S_{k+2}(\varGamma _0(N))=1$

*N*	$k+2$
1	12, 16, 18, 20, 22, 26
2	8, 10
3	6, 8
4	6
5	4, 6
6	4
7	4
8	4
9	4 (CM)
11	2
14	2
15	2
17	2
19	2
20	2
21	2
24	2
27	2 (CM)
32	2 (CM)
36	2 (CM)
49	2 (CM)

We now proceed to a proof of our main theorem relating single-valued periods to coefficients of Poincaré series in this particular case. This serves as an illustration of our proof method for the general case.

Let $f \in S_{k+2}(\varGamma _0(N);{\mathbb {Q}})$ and $g \in S^{!,\infty }_{k+2}(\varGamma _0(N);{\mathbb {Q}})$ be such that ([*f*], [*g*]) is a ${\mathbb {Q}}$-basis of $H^1_{\mathrm {dR},\mathrm {cusp}}({\mathcal {Y}}_0(N),{\mathcal {V}}_k)$ satisfying

$$\begin{aligned} \langle [f], [g] \rangle _{\mathrm {dR}} = 1\text {,} \end{aligned}$$

and write

$$\begin{aligned} \mathrm {sv}(\begin{array}{cc} [f]&[g] \end{array}) = (\begin{array}{cc} [f]&[g] \end{array})\cdot \left( \begin{array}{cc} a &{} b \\ c &{} d \end{array} \right) \end{aligned}$$

for some $a,b,c,d \in {\mathbb {R}}$. These are the single-valued periods of $H^1_{\mathrm {cusp}}({\mathcal {Y}}_0(N),V_k)$; recall from Proposition 2 that $c\ne 0$, $ad-bc=-1$, and $d=-a$. Moreover, we have

$$\begin{aligned} ([g],[f])_{\mathrm {dR}} = a\ \ \ \text { and }\ \ \ ([f],[f])_{\mathrm {dR}} = -c\text {,} \end{aligned}$$

where $( \ , \ )_{\mathrm {dR}}$ is the de Rham Hermitian form on $H^1_{\mathrm {dR},\mathrm {cusp}}({\mathcal {Y}}_0(N),{\mathcal {V}}_k)\otimes _{{\mathbb {Q}}}{\mathbb {C}}$, defined in Lemma 3 by $(\omega ,\eta )_{\mathrm {dR}} = -\langle \omega , (\mathrm {sv}\otimes c_{\mathrm {dR}})(\eta )\rangle _{\mathrm {dR}}$; see formulas (13) and (14). Set

$$\begin{aligned} \rho :=\frac{a}{c} = -\frac{([g],[f])_{\mathrm {dR}}}{([f],[f])_{\mathrm {dR}}} \in {\mathbb {R}}\text {.} \end{aligned}$$

From now on, for simplicity, we denote

$$\begin{aligned} P_{m}:=P_{m,k+2,N} \in S_{k+2}^{!,\infty }(\varGamma _0(N);{\mathbb {Q}})\text {.} \end{aligned}$$

### Proposition 8

For every positive integer *m*, there exists $h_m \in M_{-k}^{!,\infty }(\varGamma _0(N);{\mathbb {Q}})$, depending on *f* and *g*, such that, for every integer $n\ge 1$,

$$\begin{aligned} a_n(P_m) = -\frac{k!}{m^{k+1}}a_m(f)a_n(f)c^{-1}\ \ \text { and }\ \ a_n(P_{-m}) = \frac{k!}{m^{k+1}}a_m(f)a_n(f)\rho + r_{m,n} \text {,} \end{aligned}$$

where

$$\begin{aligned} r_{m,n} = \frac{k!}{m^{k+1}}a_m(f)a_n(g) + n^{k+1}a_n(h_m) \in {\mathbb {Q}}\text {.} \end{aligned}$$

In particular, $P_m$ vanishes identically if and only if $a_m(f)=0$, and in this case $a_n(P_{-m})\in {\mathbb {Q}}$ for every $n\ge 0$.

It also follows from the above proposition that ${\mathbb {Q}}(\mathrm {sv}):={\mathbb {Q}}(a,b,c,d)= {\mathbb {Q}}(c,\rho )$ coincides with the field ${\mathbb {Q}}(a_n(P_m)\text { ; }n\ge 1\text {, }m\ne 0)$ generated by the coefficients of all the weakly holomorphic Poincaré series of level *N* and weight $k+2$.

### Proof

Let *m* be a positive integer and, since $\dim S_{k+2}(\varGamma _0(N))=1$, write

30 $$\begin{aligned} P_m = \lambda f \end{aligned}$$

for some $\lambda \in {\mathbb {R}}$. By Corollary 3, we have

$$\begin{aligned} \frac{k!}{m^{k+1}}a_m(f) = ([f],[P_m])_{\mathrm {dR}} = \lambda ([f],[f])_{\mathrm {dR}} = -\lambda c \end{aligned}$$

so that

31 $$\begin{aligned} \lambda = -\frac{k!}{m^{k+1}}a_m(f)c^{-1}\text {.} \end{aligned}$$

In particular, we obtain the following general formula for the Fourier coefficients at infinity of $P_m$ in terms of the single-valued period *c*:

$$\begin{aligned} a_n(P_m) = -\frac{k!}{m^{k+1}}a_m(f) a_n(f)c^{-1}\text {.} \end{aligned}$$

Note that $P_m$ vanishes identically if and only if $a_m(f)=0$.

Now, set

32 $$\begin{aligned} f^{\flat } :=af + cg \in S_{k+2}^{!,\infty }(\varGamma _0(N);{\mathbb {R}})\text {.} \end{aligned}$$

Since $f^{\flat }$ and *f* are Betti conjugate (Definition 7), and $P_{-m}$ and $-P_m$ are Betti conjugate (Proposition 7), it follows from (30) that $-\lambda f^{\flat }$ and $P_{-m}$ map to the same class in

$$\begin{aligned} H^1_{\mathrm {dR},\mathrm {cusp}}({\mathcal {Y}}_0(N),{\mathcal {V}}_k)\otimes _{{\mathbb {Q}}}{\mathbb {R}} \cong S^{!,\infty }_{k+2}(\varGamma _0(N);{\mathbb {R}})/ D^{k+1}M_{-k}^{!,\infty }(\varGamma _0(N);{\mathbb {R}})\text {.} \end{aligned}$$

Thus, there exists $h_m\in M_{-k}^{!,\infty }(\varGamma _0(N);{\mathbb {R}})$ such that

33 $$\begin{aligned} P_{-m} = -\lambda f^{\flat } + D^{k+1}h_m\text {.} \end{aligned}$$

By considering principal parts at the cusp at infinity, we get

34 $$\begin{aligned} \frac{1}{q^m} = -\lambda {\mathcal {P}}_{f^{\flat }} + {\mathcal {P}}_{D^{k+1}h_m} {\mathop {=}\limits ^{(32)}} -\lambda c{\mathcal {P}}_g + D^{k+1}{\mathcal {P}}_{h_m} = \frac{k!}{m^{k+1}}a_m(f){\mathcal {P}}_g + D^{k+1}{\mathcal {P}}_{h_m}\text {.} \end{aligned}$$

As both *f* and *g* have rational Fourier coefficients at infinity, we get $D^{k+1}{\mathcal {P}}_{h_m} \in {\mathbb {Q}}[q^{-1}]$. Thus, ${\mathcal {P}}_{h_m} \in {\mathbb {Q}}[q^{-1}]$, and Lemma 4 implies that $h_m \in M_{-k}^{!,\infty }(\varGamma _0(N);{\mathbb {Q}})$.

Finally, by taking Fourier coefficients at infinity in (33) and applying (31), we get

$$\begin{aligned} a_n(P_{-m}) = \frac{k!}{m^{k+1}}a_m(f)a_n(f)\rho + \frac{k!}{m^{k+1}}a_m(f)a_n(g) + n^{k+1}a_n(h_m)\text {.} \end{aligned}$$

$$\square $$

When $m=1$, we can take $h_m$ to be zero. Indeed, in the above cases we always have $a_1(f)\ne 0$, so we can normalise $a_1(f)=1$. Moreover, we can assume that $\mathrm {ord}_{\infty }(g)\ge -1$, so that ${\mathcal {P}}_g = (k!)^{-1}q^{-1}$ by the condition $\langle [f],[g] \rangle _{\mathrm {dR}}=1$. Equation (34) in the above proof then implies that ${\mathcal {P}}_{h_1} = 0$, which yields $h_1=0$.

### Corollary 5

For *f* and *g* as above, we have $a_n(P_{-1}) = k!(a_n(f)\rho + a_n(g))$. $\square$

When $N=1$ and $k+2=12$, this recovers Brown’s formula ([6] Corollary 1.4), up to a difference in normalisation.

## Coefficients of Poincaré series as single-valued periods: general case

From now on, we fix an even integer $k\ge 0$, and an integer $N\ge 1$. For simplicity, we denote

$$\begin{aligned} P_m :=P_{m,k+2,N} \in S^{!,\infty }_{k+2}(\varGamma _0(N);{\mathbb {R}}) \end{aligned}$$

for every $$m \in {\mathbb {Z}}\setminus \{0\}$$.

### Proposition 9

Let $m_1,\dots ,m_s \ge 1$ be integers such that $P_{m_1},\ldots ,P_{m_s}$ generate the ${\mathbb {R}}$-vector space $S_{k+2}(\varGamma _0(N);{\mathbb {R}})$. Then, for every integer $m\ge 1$, there exist $\lambda _1,\ldots ,\lambda _s \in {\mathbb {Q}}$ and $h \in M_{-k}^{!,\infty }(\varGamma _0(N);{\mathbb {Q}})$ such that

$$\begin{aligned} P_m = \lambda _1P_{m_1} + \cdots + \lambda _s P_{m_s} \end{aligned}$$

and

$$\begin{aligned} P_{-m} = \lambda _1 P_{-m_1} + \cdots + \lambda _s P_{-m_s} + D^{k+1}h\text {.} \end{aligned}$$

### Proof

For every integer $m\ge 1$, recall that we view $f\mapsto a_m(f)$ as a linear functional on $S_{k+2}(\varGamma _0(N))$; it follows from Corollary 3 that

35 $$\begin{aligned} ( \ \ , [P_m])_{\mathrm {dR}} = \frac{k!}{m^{k+1}}a_m \end{aligned}$$

in $S_{k+2}(\varGamma _0(N))^{\vee }$. Since $P_{m_1},\ldots ,P_{m_s}$ generate $S_{k+2}(\varGamma _0(N);{\mathbb {R}})$, and since $( \ , \ )_{\mathrm {dR}}$ is non-degenerate on $S_{k+2}(\varGamma _0(N))$, the functionals $a_{m_1},\ldots ,a_{m_s}$ generate the ${\mathbb {Q}}$-vector space $S_{k+2}(\varGamma _0(N);{\mathbb {Q}})^{\vee }$. Thus, there exist $\lambda _1,\ldots ,\lambda _s \in {\mathbb {Q}}$ such that $a_m = \lambda _1 a_{m_1} + \cdots + \lambda _s a_{m_s}$. Again, by (35) and by the non-degeneracy of $( \ , \ )_{\mathrm {dR}}$, we conclude that

$$\begin{aligned} P_m = \lambda _1 P_{m_1} + \cdots + \lambda _s P_{m_s}\text {.} \end{aligned}$$

Now, Proposition 7 implies that both $P_{-m}$ and $\lambda _1P_{-m_1} + \cdots + \lambda _s P_{-m_s}$ are Betti conjugate to $-P_m$; in particular, they map to the same class in $H^1_{\mathrm {dR}}({\mathcal {Y}}_0(N),{\mathcal {V}}_k)\otimes _{{\mathbb {Q}}}{\mathbb {R}}$. Thus, by Corollary 2, there exists $h\in M^{!,\infty }_{-k}(\varGamma _0(N);{\mathbb {R}})$ such that

$$\begin{aligned} P_{-m} = \lambda _1P_{-m_1} + \cdots + \lambda _s P_{-m_s} + D^{k+1}h\text {.} \end{aligned}$$

Finally, since $\lambda _i \in {\mathbb {Q}}$, by taking principal parts at the cusp at infinity, we deduce from Proposition 3 and Lemma 4 that $h \in M_{-k}^{!,\infty }(\varGamma _0(N);\mathbb {{\mathbb {Q}}})$. $\square$

### Corollary 6

Let $m_1,\dots ,m_s \ge 1$ be integers such that $P_{m_1},\ldots ,P_{m_s}$ generate the ${\mathbb {R}}$-vector space $S_{k+2}(\varGamma _0(N);{\mathbb {R}})$. Then, for every integers $m,n\ge 1$,

1. $a_n(P_{m})$ lies in the ${\mathbb {Q}}$-linear span of $\{a_n(P_{m_i}) \text { ; } 1\le i \le s\}$, and
2. $a_n(P_{-m})$ lies in the ${\mathbb {Q}}$-linear span of $\{1\}\cup \{a_n(P_{-m_i}) \text { ; } 1 \le i \le s\}$.

### Proof

Consider Fourier coefficients at infinity in the statement of Proposition 9. $\square$

We come to our main theorem.

### Theorem 8

Let ${\mathbb {Q}}(\mathrm {sv})\subset {\mathbb {R}}$ be the field of rationality of the single-valued involution

$$\begin{aligned} \mathrm {sv}: H^1_{\mathrm {dR},\mathrm {cusp}}({\mathcal {Y}}_0(N),{\mathcal {V}}_k)\otimes _{{\mathbb {Q}}}{\mathbb {R}} \rightarrow H^1_{\mathrm {dR},\mathrm {cusp}}({\mathcal {Y}}_0(N),{\mathcal {V}}_k)\otimes _{{\mathbb {Q}}}{\mathbb {R}}\text {,} \end{aligned}$$

and ${\mathbb {Q}}(P)\subset {\mathbb {R}}$ be the ${\mathbb {Q}}$-extension generated by the Fourier coefficients at infinity of all the Poincaré series $P_m$, for $m\in {\mathbb {Z}}\setminus \{0\}$. Then,

$$\begin{aligned} {\mathbb {Q}}(\mathrm {sv}) = {\mathbb {Q}}(P)\text {.} \end{aligned}$$

### Proof

Let $d = \dim S_{k+2}(\varGamma _0(N))$. We showed in Sect. 6 that the object $H_{\mathrm {cusp}}^1({\mathcal {Y}}_0(N),V_k)$ of ${\mathcal {H}}({\mathbb {Q}})$ is pure of Hodge type $\{(k+1,0),(0,k+1)\}$ and admits a polarisation $\langle \ , \ \rangle$. In particular, by Corollary 2, there exist $f_1,\ldots ,f_d,g_1,\ldots ,g_d \in S_{k+2}^{!,\infty }(\varGamma _0(N);{\mathbb {Q}})$ such that

$$\begin{aligned} b_{\mathrm {dR}} = ([f_1],\ldots ,[f_d],[g_1],\ldots ,[g_d]) \end{aligned}$$

is a ${\mathbb {Q}}$-basis of $H^1_{\mathrm {dR},\mathrm {cusp}}({\mathcal {Y}}_0(N),{\mathcal {V}}_k)$ as in (12), that is,

- $([f_1],\ldots ,[f_d])$ is a ${\mathbb {Q}}$-basis of $F_{\mathrm {dR}}^{k+1}H^1_{\mathrm {dR},\mathrm {cusp}}({\mathcal {Y}}_0(N),{\mathcal {V}}_k)$ (or, equivalently, $(f_1,\ldots ,f_d)$ is a ${\mathbb {Q}}$-basis of $S_{k+2}(\varGamma _0(N);{\mathbb {Q}})$), and
- $\langle [f_i], [g_j] \rangle _{\mathrm {dR}} = \delta _{ij}$ and $\langle [g_i],[g_j] \rangle _{\mathrm {dR}} = 0$ for every $1\le i,j\le d$.

We prove first that ${\mathbb {Q}}(P)\subset {\mathbb {Q}}(\mathrm {sv})$. For this, let $m_1,\ldots ,m_d\ge 1$ be integers such that $(P_{m_1},\ldots ,P_{m_d})$ is an ${\mathbb {R}}$-basis of $S_{k+2}(\varGamma _0(N);{\mathbb {R}})$, and write

$$\begin{aligned} S = \left( \begin{array}{cc} A &{} B \\ C &{} D \end{array}\right) \in {\mathrm {GL}}_{2g}({\mathbb {R}}) \end{aligned}$$

for the matrix of the single-valued involution $\mathrm {sv}$ in the basis $b_{\mathrm {dR}}$. By Corollary 6, it suffices to prove that $a_n(P_{ m_i}), a_n(P_{-m_i})\in {\mathbb {Q}}(S)$ for every integer $n\ge 1$ and $1\le i \le d$. Let $\varLambda = (\varLambda _{ij})_{i,j} \in {\mathrm {GL}}_d({\mathbb {R}})$ be such that

36 $$\begin{aligned} P_{m_j} = \sum _{i=1}^d\varLambda _{ij}f_i\text {, }\qquad 1\le j \le d\text {.} \end{aligned}$$

By applying Corollary 3 in the above formula, we obtain

$$\begin{aligned} \frac{k!}{m_j^{k+1}}a_{m_j}(f_r) = ([f_r],[P_{m_j}])_{\mathrm {dR}} = \sum _{i=1}^d([f_r],[f_i])_{\mathrm {dR}}\varLambda _{ij} = -\sum _{i=1}^dC_{ri}\varLambda _{ij}\text {,} \end{aligned}$$

where we used the identity (13) in the last equality above. Set

$$\begin{aligned} Q :=(\frac{k!}{m_j^{k+1}}a_{m_j}(f_r))_{r,j} \in \mathrm {GL}_{d}({\mathbb {Q}})\text {.} \end{aligned}$$

The above equations amount to the matrix identity in $\mathrm {GL}_d({\mathbb {R}})$

37 $$\begin{aligned} \varLambda = -C^{-1}Q\text {.} \end{aligned}$$

Since *Q* has rational coefficients, this proves already that $a_n(P_{m_j}) \in {\mathbb {Q}}(C) \subset {\mathbb {Q}}(S)$ for every $n\ge 1$ and $1\le j \le d$.

For every $1\le j \le d$, set

$$\begin{aligned} f^{\flat }_j :=\sum ^d_{i=1}A_{ij}f_i + C_{ij}g_i \in S^{!,\infty }_{k+2}(\varGamma _0(N);{\mathbb {Q}}(\mathrm {sv}))\text {.} \end{aligned}$$

By definition of *S*, $f_j^{\flat }$ is Betti conjugate to $f_j$. Then, it follows from Proposition 7 and from (36) that both $P_{-m_j}$ and $- \sum _{i=1}^d \varLambda _{ij}f^{\flat }_i$ are Betti conjugate to $-P_{m_j}$. Thus, by Corollary 2, there exists $h_j \in M_{-k}^{!,\infty }(\varGamma _0(N))$ such that

38 $$\begin{aligned} P_{-m_j} = - \sum _{i=1}^d \varLambda _{ij}f^{\flat }_i + D^{k+1}h_j\text {.} \end{aligned}$$

By taking principal parts at the cusp at infinity in the above equation, and by applying Lemma 4 and (37), we get

$$\begin{aligned} h_j \in M_{-k}^{!,\infty }(\varGamma _0(N); {\mathbb {Q}}(\varLambda ))= M_{-k}^{!,\infty }(\varGamma _0(N); {\mathbb {Q}}(C))\text {.} \end{aligned}$$

Finally, by taking Fourier coefficients at infinity in Eq. (38), we obtain

$$\begin{aligned} a_n(P_{-m_j}) \in {\mathbb {Q}}(A,C) \subset {\mathbb {Q}}(S) \end{aligned}$$

for every integer $n\ge 1$. This concludes the proof that ${\mathbb {Q}}(P)\subset {\mathbb {Q}}(S)$.

For the reverse inclusion, note that Proposition 2 yields ${\mathbb {Q}}(\mathrm {sv}) = {\mathbb {Q}}(A,C)$. The formulas (36) and (37) above imply that ${\mathbb {Q}}(C)\subset {\mathbb {Q}}(P)$; thus, to finish our proof, it suffices to show that ${\mathbb {Q}}(A) \subset {\mathbb {Q}}(P)$.

If we denote $\varLambda ' = \varLambda ^{-1} \in \mathrm {GL}_{d}({\mathbb {Q}}(C))$, then we can write

$$\begin{aligned} f_{j} = \sum _{i=1}^d\varLambda '_{ij}P_{m_i}\text {,}\qquad 1\le j \le d\text {.} \end{aligned}$$

Arguing as above, we deduce that both $f^{\flat }_j$ and $-\sum _{i=1}^d \varLambda _{ij}'P_{-m_i}$ are Betti conjugate to $f_j$, so that there exists $h_j' \in M_{-k}^{!,\infty }(\varGamma _0(N);{\mathbb {Q}}(C))$ such that

$$\begin{aligned} \sum _{i=1}^d A_{ij}f_i + C_{ij}g_i = -\sum _{i=1}^d\varLambda _{ij}'P_{-m_i} + D^{k+1}h_j'\text {.} \end{aligned}$$

By applying the functionals $\frac{k!}{m_r^{k+1}}a_{m_r}$ to the above equation, we get $Q^tA = M$, for some $M \in M_{d\times d}({\mathbb {Q}}(P))$ (recall that ${\mathbb {Q}}(C)\subset {\mathbb {Q}}(P)$). Here, *Q* is the same matrix of Eq. (36); since it is invertible and has rational coefficients, we conclude that ${\mathbb {Q}}(A)\subset {\mathbb {Q}}(P)$. $\square$

## Hecke theory; single-valued periods of modular forms

In this last section, we explain how to use Hecke operators to obtain simple formulas, similar to the rank 2 case, relating the single-valued periods of $H^1_{\mathrm {cusp}}({\mathcal {Y}}_0(N),V_k)$ to the Fourier coefficients of Poincaré series of weight $k+2$.

Recall that, for every prime $p\not \mid N$, there is a correspondence 
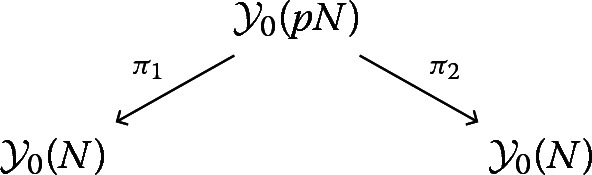
 defined by $\pi _1 (E,C) = (E,C_N)$ and $\pi _2(E,C) = (E/C_p,C/C_p)$, where $C_N$ and $C_p$ denote the unique cyclic subgroups of *C* of order *N* and *p*. This induces an endomorphism $T_p$ (Hecke operator) of $H^1({\mathcal {Y}}_0(N),V_k)$ in ${\mathcal {H}}({\mathbb {Q}})$ by the usual pullback–pushforward procedure (see [14] (3.13)–(3.18) for details).

Given $f \in M_{k+2}^!(\varGamma _0(N))$, we have $T_{p,\mathrm {dR}}[f] = [T_pf]$, where

$$\begin{aligned} T_pf :=\sum _{n\ge 1}(a_{pn}(f) + p^{k+1}a_{n/p}(f))q^n\text {.} \end{aligned}$$

By construction, the induced endomorphism $T_p$ on $H^1_{\mathrm {cusp}}({\mathcal {Y}}_0(N),V_k)$ can be shown to be compatible with the polarisation $\langle \ , \ \rangle$, that is, $\langle T_p \ , \ \rangle = \langle \ , T_p \ \rangle$.

Let $f \in S_{k+2}(\varGamma _0(N))$ be a normalised Hecke newform (in particular, $a_1(f)=1$ and $T_pf = a_p(f)f$ for every prime $p\not \mid N$). Recall that the subfield $K_f :={\mathbb {Q}}(a_n(f) \text { ; } n\ge 1)\subset {\mathbb {C}}$ is totally real ([12] Proposition 10.6.2).

### Proposition 10

There exists a unique rank 2 polarised subobject $H_f$ of $H_{\mathrm {cusp}}^1({\mathcal {Y}}_0(N),V_k)\otimes _{{\mathbb {Q}}}K_f$ in ${\mathcal {H}}(K_f)$ given by $\bigcap _{p\not \mid N}\ker (T_p-a_p(f)\mathrm {id})$.

### Proof

This follows from the ‘multiplicity 1 theorem’ ([12] Theorem 13.3.9) and can be checked directly on the de Rham and Betti realisations. See also [35]. $\square$

Concretely, the de Rham realisation $H_{f,\mathrm {dR}}$ is a $K_f$-vector space of dimension 2 admitting a basis of the form ([*f*], [*g*]), with $g \in S_{k+2}^{!,\infty }(\varGamma _0(N);K_f)$ satisfying

1. $$\langle [f],[g]\rangle _{\mathrm {dR}}=1$$, and
2. for every prime $p\not \mid N$, we have $T_pg =a_p(f)g + D^{k+1}h_{p}$, for some $h_p \in M_{-k}^{!,\infty }(\varGamma _0(N);K_f)$.

We now wish to express the single-valued periods of $H_f$ in terms of coefficients of Poincaré series. To simplify, we shall assume from now on that

$$\begin{aligned} S_{k+2}(\varGamma _0(N)) = S_{k+2}^{\text {new}}(\varGamma _0(N))\text {.} \end{aligned}$$

This happens, for instance, when $N=1$, or when *N* is prime and $S_{k+2}(\varGamma _0(1))=0$. In this case, there exists a unique basis $(f_1,\ldots ,f_d)$ of $S_{k+2}(\varGamma _0(N))$, up to ordering, where each $f_i$ is a normalised Hecke newform. If $K\subset {\mathbb {R}}$ is the compositum of $K_{f_1},\ldots ,K_{f_d}$, then this basis induces a splitting

$$\begin{aligned} H^1_{\mathrm {cusp}}({\mathcal {Y}}_0(N),V_k)\otimes _{{\mathbb {Q}}}K \cong \bigoplus _{i=1}^dH_{f_i}\otimes _{K_{f_i}}K \end{aligned}$$

in ${\mathcal {H}}(K)$. Note that such a decomposition is necessarily orthogonal for $\langle \ , \ \rangle _{\mathrm {dR}}$, since, for $\omega _i \in H_{f_i,\mathrm {dR}}$ and $\omega _j \in H_{f_j,\mathrm {dR}}$, we have

$$\begin{aligned} a_p(f_i)\langle \omega _i,\omega _j\rangle _{\mathrm {dR}} = \langle T_{p,\mathrm {dR}}\omega _i,\omega _j\rangle _{\mathrm {dR}} = \langle \omega _i,T_{p,\mathrm {dR}}\omega _j\rangle _{\mathrm {dR}} = a_p(f_j)\langle \omega _i,\omega _j\rangle _{\mathrm {dR}} \end{aligned}$$

for every prime $p\not \mid N$.

For each *i*, let $g_i \in S_{k+2}^{!,\infty }(\varGamma _0(N);K_{f_i})$ be a weakly holomorphic modular form satisfying properties (1) and (2) above. We denote the single-valued period matrix of $H_{f_i}$ in the basis $([f_i],[g_i])$ by

$$\begin{aligned} S_i = \left( \begin{array}{cc} a_i &{} b_i\\ c_i &{} d_i \end{array}\right) \end{aligned}$$

Then, $c_i\ne 0$ and we set $\rho _i :=a_i/c_i$.

### Theorem 9

For every integer $m\ge 1$, there exists $h_m \in M_{-k}^{!,\infty }(\varGamma _0(N);K)$ such that

$$\begin{aligned} P_m = -\frac{k!}{m^{k+1}}\sum _{i=1}^d a_{m}(f_i)c_i^{-1}f_i \end{aligned}$$

and

$$\begin{aligned} P_{-m} = \frac{k!}{m^{k+1}}\left( \sum _{i=1}^d a_{m}(f_i)\rho _i f_i + a_{m}(f_i)g_i \right) + D^{k+1}h_m\text {.} \end{aligned}$$

### Proof

Let $\lambda _i \in {\mathbb {R}}$ be such that $P_m = \sum _{i=1}^d\lambda _i f_i$. Since the $f_i$ are orthogonal for $( \ , \ )_{\mathrm {dR}}$, Corollary 3 yields

$$\begin{aligned} \frac{k!}{m^{k+1}}a_m(f_i) = ([f_i],[P_m])_{\mathrm {dR}} = \sum _{j=1}^d\lambda _j([f_i],[f_j])_{\mathrm {dR}} = \lambda _i([f_i],[f_i])_{\mathrm {dR}} = -c_i \lambda _i\text {.} \end{aligned}$$

This proves the first formula. By Proposition 7, $P_{-m}$ and $-\sum _{i=1}^{d}\lambda _i(a_if_i+c_ig_i)$ are Betti conjugate, so that by Corollary 2 there exists $h_m \in M_{-k}^{!,\infty }(\varGamma _0(N))$ such that

$$\begin{aligned} P_{-m} = \sum _{i=1}^d \lambda _i(a_if_i+c_ig_i) + D^{k+1}h_m\text {.} \end{aligned}$$

By taking principal parts at infinity and applying Lemma 4, we conclude that the Fourier coefficients at infinity of $h_m$ lie in *K*. $\square$

Taking a basis of Poincaré series, we can invert the above formulas.

### Theorem 10

Let $m_1,\ldots ,m_d\ge 1$ be integers such that $(P_{m_1},\ldots ,P_{m_d})$ is a basis of $S_{k+2}(\varGamma _0(N))$, and let $(r_{ij}) = (a_{m_j}(f_i))^{-1} \in \mathrm {GL}_{d}(K)$. Then, for every $1\le j \le d$, there exists $h_j' \in M_{-k}^{!,\infty }(\varGamma _0(N);K)$ such that

$$\begin{aligned} -c_j^{-1}f_j = \frac{1}{k!}\sum _{i=1}^dm_i^{k+1}r_{ij} P_{m_i} \end{aligned}$$

and

$$\begin{aligned} \rho _j f_j + g_j = \frac{1}{k!}\left( \sum _{i=1}^dm_i^{k+1}r_{ij}P_{-m_i}\right) + D^{k+1}h'_{j}\text {.} \end{aligned}$$

### Proof

Let $(\lambda _{ij}) \in \mathrm {GL}_{d}({\mathbb {R}})$ be such that $P_{m_j} = \sum _{i=1}^d\lambda _{ij} f_i$. It follows from Theorem 9 that $\lambda _{ij} = -k!c_i^{-1}a_{m_j}(f_i)m_j^{-k-1}$. In matrix notation,

$$\begin{aligned} \left( \begin{array}{ccc} &{} &{} \\ &{} \lambda _{ij} &{} \\ &{} &{} \end{array} \right) = -k!\left( \begin{array}{ccc} c_1&{} &{} \\ &{} \ddots &{} \\ &{} &{} c_d \end{array} \right) ^{-1}\left( \begin{array}{ccc} &{} &{} \\ &{} a_{m_j}(f_i) &{} \\ &{} &{} \end{array} \right) \left( \begin{array}{ccc} m_1 &{} &{} \\ &{} \ddots &{} \\ &{} &{} m_d \end{array} \right) ^{-k-1}\text {.} \end{aligned}$$

Thus, $f_j = -\frac{1}{k!}\sum _{i=1}^dm_{i}^{k+1}r_{ij}c_jP_{m_i}$ and our first formula follows. By Proposition 7, $\rho _jf_j + g_j$ and $\sum _{i=1}^{d}m_{i}^{k+1}r_{ij}P_{-m_i}$ are Betti conjugate, so that by Corollary 2 there exists $h_j' \in M_{-k}^{!,\infty }(\varGamma _0(N))$ such that

$$\begin{aligned} \rho _j f_j + g_j = \frac{1}{k!}\left( \sum _{i=1}^dm_i^{k+1}r_{ij}P_{-m_i}\right) + D^{k+1}h'_{j}\text {.} \end{aligned}$$

By taking principal parts at infinity and applying Lemma 4, we conclude that the Fourier coefficients at infinity of $h'_j$ lie in *K*. $\square$

We can always choose $g_j$ such that $a_1(g_j)=0$. In particular, by Proposition 4, we obtain

39 $$\begin{aligned} \rho _j =\frac{2\pi (-1)^{\frac{k+2}{2}}}{k!}\sum _{c\ge 1\text {, } N \mid c}\left( \sum _{i=1}^d m_i^{\frac{k+1}{2}}r_{ij}\frac{K(-m_i,1;c)}{c}I_{k+1}\left( \frac{4\pi \sqrt{m_i}}{c}\right) \right) + a_1(h_j')\text {.} \end{aligned}$$

Let us also remark that, once the classical periods $\omega ^+_j, \omega ^-_j$ of $f_j$ are known, the entire period matrix

$$\begin{aligned} P_j = \left( \begin{array}{cc} \omega ^+_j &{} \eta ^+_j\\ i\omega ^-_j &{} i\eta ^-_j \end{array}\right) \end{aligned}$$

of $H_{f_j}$ is determined by $\omega ^+_j,\omega ^-_j,\rho _j$, since one always has the relation $\det P_j \in {\mathbb {Q}}^{\times }(2\pi i)^{k+1}$. As a result, we can obtain ‘universal’ expressions for the quasi-periods of Hecke eigenforms (in the terminology of [8]) in terms of explicit series involving Kloosterman sums and special values of Bessel functions.

## Footnotes

1 Deciding whether a Fourier coefficient of a Poincaré series is nonzero is a hard problem in general. For instance, Lehmer’s conjecture on the non-vanishing of Ramanujan’s $\tau$ function is equivalent to the assertion that $P_{m,12,1}$ does not vanish identically for every $m>0$.

2 This also follows the above geometric description of $M_r(\varGamma _0(N);K)$, since the Hodge line bundle $\overline{{\mathcal {F}}}$ is ample over a finite étale cover *X* of ${\mathcal {X}}_0(N)$ ([18] VII.3.4).
